# Oxidative stress promotes fibrosis in systemic sclerosis through stabilization of a kinase-phosphatase complex

**DOI:** 10.1172/jci.insight.155761

**Published:** 2022-04-22

**Authors:** Ruiyuan Zhang, Ganesan Senthil Kumar, Uwe Hansen, Martina Zoccheddu, Cristiano Sacchetti, Zachary J. Holmes, Megan C. Lee, Denise Beckmann, Yutao Wen, Zbigniew Mikulski, Shen Yang, Eugenio Santelli, Rebecca Page, Francesco Boin, Wolfgang Peti, Nunzio Bottini

**Affiliations:** 1 Department of Medicine and; 2 Department of Biological Sciences, University of California, San Diego, La Jolla, California, USA.; 3 Department of Molecular Biology & Biophysics, University of Connecticut Health Center, Farmington, Connecticut, USA.; 4 Institute for Musculoskeletal Medicine, University of Munster, Munster, Germany.; 5 Microscopy and Histology Core Facility, La Jolla Institute for Immunology, La Jolla, California, USA.; 6 Department of Cell Biology, University of Connecticut Health Center, Farmington, Connecticut, USA.; 7 Division of Rheumatology, Cedars-Sinai Medical Center, Los Angeles, California, USA.; 8 Department of Medicine, University of California, San Francisco, San Francisco, California, USA.

**Keywords:** Inflammation, Autoimmune diseases, Fibrosis, Rheumatology

## Abstract

Systemic sclerosis (SSc) is a fibrotic autoimmune disease characterized by pathogenic activation of fibroblasts enhanced by local oxidative stress. The tyrosine phosphatase PTP4A1 was identified as a critical promoter of TGF-β signaling in SSc. Oxidative stress is known to functionally inactivate tyrosine phosphatases. Here, we assessed whether oxidation of PTP4A1 modulates its profibrotic action and found that PTP4A1 forms a complex with the kinase SRC in scleroderma fibroblasts, but surprisingly, oxidative stress enhanced rather than reduced PTP4A1’s association with SRC and its profibrotic action. Through structural assessment of the oxo-PTP4A1-SRC complex, we unraveled an unexpected mechanism whereby oxidation of a tyrosine phosphatase promotes its function through modification of its protein complex. Considering the importance of oxidative stress in the pathogenesis of SSc and fibrosis, our findings suggest routes for leveraging PTP4A1 oxidation as a potential strategy for developing antifibrotic agents.

## Graphical abstract

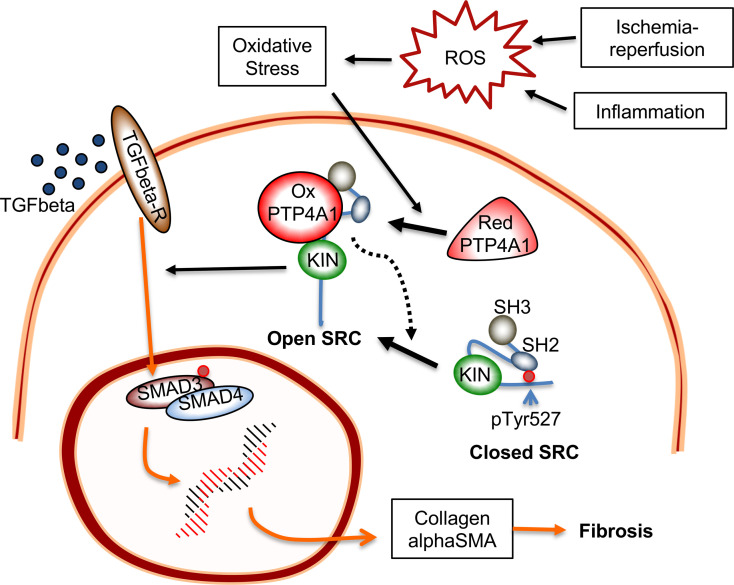

## Introduction

Systemic sclerosis (SSc) is an autoimmune disease characterized by progressive fibrosis of the skin and internal organs, with significant morbidity and mortality (1). Protein tyrosine kinases (PTKs) are key promoters of profibrotic signaling and validated drug targets in SSc (2). The actions of PTKs are counteracted by protein tyrosine phosphatases (PTPs), but unlike PTKs, PTPs have only recently emerged as viable drug targets (3).

Oxidative stress is critically involved in the pathogenesis of SSc (4). SSc dermal fibroblasts (SScDFs) have higher expression/activity of NADPH oxidase and produce higher levels of ROS compared with normal human dermal fibroblasts (NHDFs), which is further enhanced by profibrotic TGF-β (4, 5). The membrane-permeable antioxidant N-acetyl-cysteine (NAC) inhibits collagen deposition by SScDFs and has been tested in trials of SSc fibrosis with some benefit (4). A key hypothesized mechanism of growth factor–ROS interplay in signal transduction is through catalytic and functional inactivation of Cys-based inhibitory PTPs via oxidation of their catalytic Cys (6). However, besides a single report documenting that oxidative inactivation of PTP1B enhances profibrotic signaling in SSc fibroblasts (7), it remains unknown whether other PTPs are regulated by oxidative stress in SSc.

SRC kinase has been identified as a key promoter of profibrotic signaling in SSc fibroblasts (2). SRC belongs to a large family of cytoplasmic PTKs that share a common architecture, namely a kinase domain, a preceding regulatory module composed of an SH3 and an SH2 (for SRC-homology 3 and 2) domain, an N-terminal tail of largely unknown function, and a short regulatory C-terminal tail (8, 9). The activity of SRC is regulated by 2 major phosphorylation events on tyrosine residues Y^416^ (activation loop) and Y^527^ (C-terminal tail; chicken SRC numbering), which have opposing effects on SRC activity. When SRC Y^527^ is phosphorylated by C-terminal SRC kinase (CSK), pY^527^containing C-terminal tail wraps around the kinase domain to bind the N-terminal SH2 domain, thereby stabilizing SRC in a closed conformation. This remodels the activation loop, rendering the enzyme inactive (10, 11). In contrast, phosphorylation of Y^416^ leads to the release of the activation loop, resulting in increased SRC kinase activity (12, 13). Phosphorylation of Y^416^ also promotes SRC ubiquitination and subsequent degradation (14).

We recently reported that protein tyrosine phosphatase 4A type 1 (PTP4A1) is overexpressed in dermal fibroblasts and myofibroblasts from patients with SSc, and it supports sustained TGF-β signaling by enhancing SRC activity (15). PTP4A1 belongs to a family of 3 Cys-based PTPs, PTP4A1–3, which have recently attracted attention as Mg^2+^ level regulators via an interaction with the cyclin and cystathionine β-synthase domain magnesium transport mediators (CNNMs) (16, 17). PTP4A phosphatases also undergo reversible oxidative inactivation via formation of a disulfide bond between the catalytic Cys (Cys^104^ in PTP4A1) and a second spatially close Cys (Cys^49^ in PTP4A1) residue (17, 18).

Here, we sought to understand the regulation of PTP4A1 function by oxidative stress in SScDFs. We initially expected that oxidation of PTP4A1 would remove its SRC-promoting effect, potentially playing a protective role in SSc fibrosis. Surprisingly, we instead found that oxidation of PTP4A1 promoted its association with SRC, and we unraveled a mechanism whereby oxidative stress promoted the function of a PTP despite causing its catalytic inactivation.

## Results

### PTP4A1 and SRC form a complex in SScDFs.

**Figure 1:**
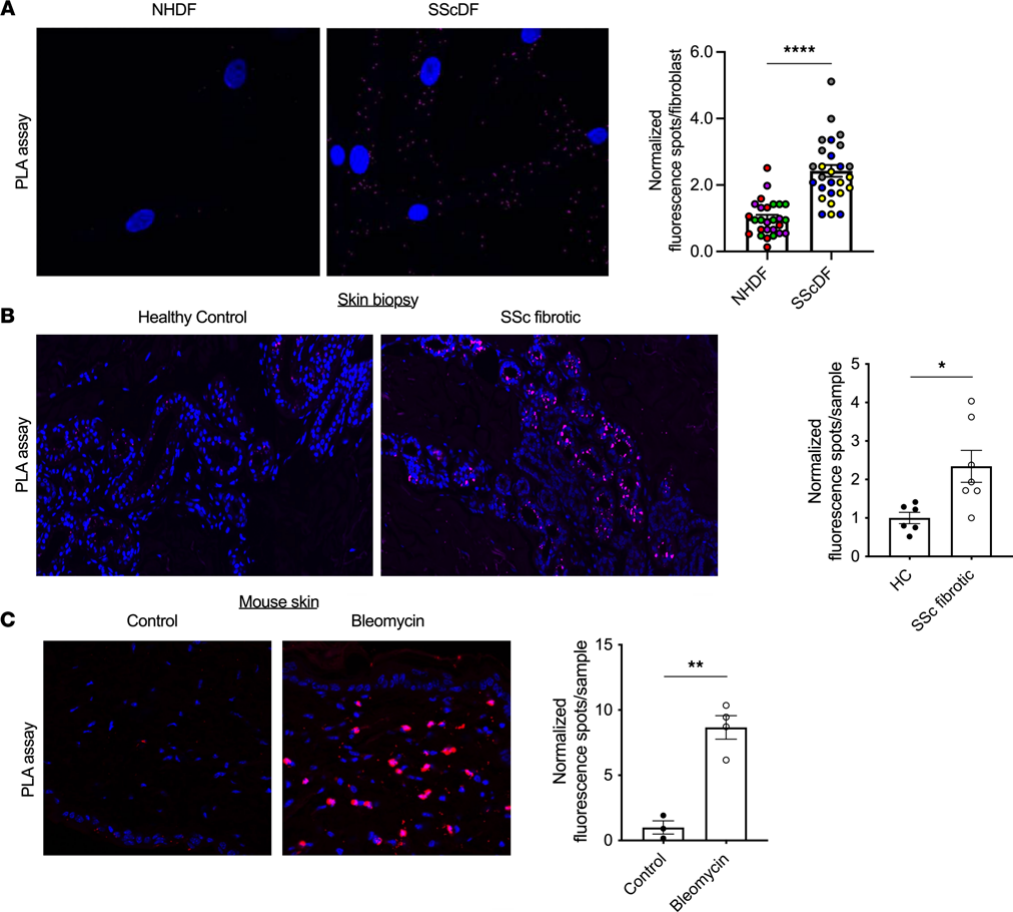
PTP4A1 and SRC form a complex in dermal fibroblasts. (**A**) Representative PTP4A1-SRC PLA signal in NHDFs versus SScDFs (left) with quantification (right). *n* = 3 cell lines each. Dots of the same color are from the same NHDF or SScDF cell line. (**B**) Representative PTP4A1-SRC PLA signal in skin specimens from healthy donors (*n* = 6) versus fibrotic patients with SSc (*n* = 7) (left) with quantification (right). Each point represents an individual donor. (**C**) Representative PLA signal in skin of mice treated with bleomycin (*n* = 4) or control mice (*n* = 3) (left) with quantification (right). (**A** and **B**) Blue = DAPI, magenta = PLA. (**C**) Blue = DAPI, red = PLA, magenta = overlap. (**A**) Images were captured with 60× original magnification. (**B** and **C**) Images were captured with 20× original magnification. (**A**–**C**) Data shown are mean ± SEM of data normalized to the control averages. Two-tailed Welch’s *t* test. *P < 0.05, **P < 0.01, ****P < 0.0001.

We previously proposed that PTP4A1 (but not PTP4A2; ref. 15) regulates SRC activation in TGF-β–stimulated fibroblasts via formation of a PTP4A1-SRC complex. However, direct evidence of an SRC-PTP4A1 interaction in cells, elucidation of its molecular basis, and potential mechanisms for its regulation are still lacking. We thus decided to examine SRC-PTP4A1 complex formation in cells through proximity ligation assays (PLAs) (19). As shown in Supplemental Figure 1 (supplemental material available online with this article; https://doi.org/10.1172/jci.insight.155761DS1), NHDFs stimulated with TGF-β displayed significantly more PLA fluorescence signal compared with unstimulated cells, correlating with increased expression of PTP4A1. The anti-PTP4A1 Ab used here reacts with all PTP4A proteins, and NHDFs and SScDFs express both PTP4A1 and PTP4A2, while PTP4A3 is undetectable (15). Therefore, to confirm that our PLA assay primarily detects a PTP4A1-SRC complex, we performed the same assay in NHDFs overexpressing either protein and showed that NHDFs overexpressing PTP4A1 exhibited more PLA signal compared with the same cells overexpressing PTP4A2. Furthermore, in TGF-β–stimulated NHDFs, the PLA signal was virtually abolished by PTP4A1 but not PTP4A2 knockdown (15) (Supplemental Figure 2). The lack of a signal due to PTP4A2 is consistent with our previous data that PTP4A2 only weakly coprecipitates with SRC in HEK293T cells compared with PTP4A1. We then assessed the interaction between PTP4A1 and SRC in SSc-relevant contexts. Using the same assay, we found that PLA-positive spots were significantly more abundant in SScDFs compared with NHDFs (Figure 1A). After adapting the PLA assay to paraffin-embedded specimens to detect the complex between PTP4A1 and SRC in tissues, we also found a higher PLA signal in the dermis of patients with SSc versus healthy controls (Figure 1B). Likewise, the PLA signal was elevated in the skin of mice with bleomycin-induced fibrosis, a model that recapitulates the combined action of growth factors and oxidative stress in SSc (Figure 1C) (20), compared with nonfibrotic untreated mice.

### SRC preferentially binds the oxidized form of PTP4A1.

**Figure 2:**
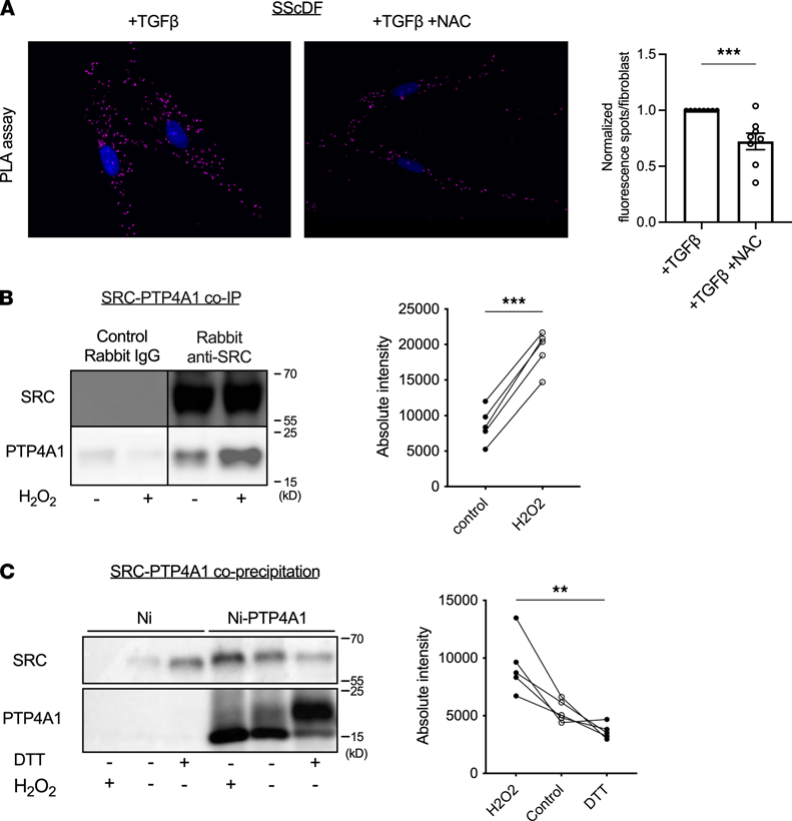
Oxidative stress promotes PTP4A1 and SRC association. (**A**) Representative PTP4A1-SRC PLA signal in TGF-β–stimulated SScDFs treated with or without NAC (left) with quantification (right). *n* = 8 experiments across 7 cell lines. (**B**) Representative Western blotting image of co-IP between PTP4A1 and SRC from HEK293T cells untreated or incubated with H_2_O_2_ (top) with quantification (bottom). *n* = 5, reducing buffer. Rabbit IgG was used for control IP. (**C**) Binding of SRC to Ni-NTA agarose-bound His_6_-tagged oxidized/reduced PTP4A1. Ni-NTA agarose beads were used as control. Non-reducing buffer. (**A**) Blue = DAPI, magenta = PLA. (**A**) Images were captured with 40× original magnification. Data shown are mean ± SEM of data normalized to the control averages. Two-tailed paired *t* test from the non-normalized data in **A**, 2-tailed Welch’s *t* test in **B**, 1-way ANOVA in **C**. **P < 0.01, ***P < 0.001.

PTP4A proteins are especially stable in their oxidized form, and oxidation inactivates their phosphatase activity (18) and impairs their interaction with CNNM channels (17). We therefore explored whether PTP4A1 oxidation could modulate its profibrotic action and began by investigating the effect of an oxidative versus reducing environment on PTP4A1-SRC complex formation in cells. Since SScDFs have high levels of endogenous ROS production and PTP inactivation (4, 7), we first incubated TGF-β–stimulated SScDFs with NAC, expecting it to rescue PTP4A1 activity and enhance PTP4A1-SRC complex formation. Surprisingly, we observed a reduced PLA signal in NAC-treated cells, suggesting that PTP4A1-SRC interaction was enhanced under conditions of oxidative stress (Figure 2A and Supplemental Figure 3). Consistent with this observation, incubation with 100 μM H_2_O_2_ for 30 minutes, sufficient to fully oxidize PTP4A1 (Supplemental Figure 4), strongly enhanced co-IP of PTP4A1 with SRC in HEK293T cells (Figure 2B) (15). In a similar assay using in vitro oxidized/reduced SRC and PTP4A1 isolated from bacteria, significantly more SRC was precipitated by oxidized than reduced PTP4A1 (Figure 2C), while untreated beads containing both oxidized and reduced PTP4A1 displayed intermediate binding. Together, these data demonstrate a specific interaction between oxidized PTP4A1 and SRC in vitro and in cells and suggest that oxidation of PTP4A1 by endogenous or exogenous ROS contributes to ROS-induced amplification of profibrotic signaling in SScDFs.

### oxo-PTP4A1 binds SRC through its active site.

**Figure 3:**
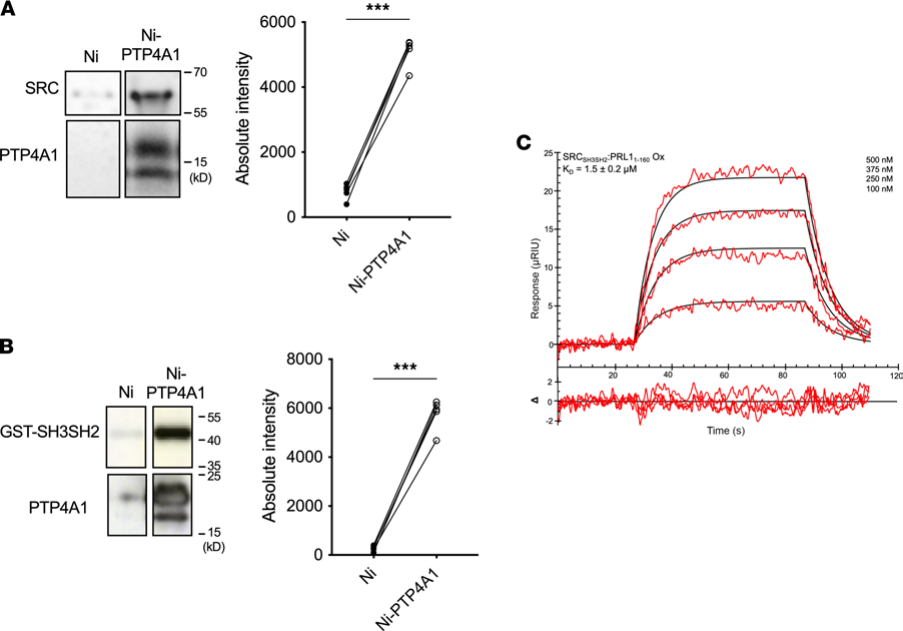
PTP4A1 binds SRC on the SH3SH2 domains. (**A** and **B**) Representative Western blotting (left) with quantification (right) of (**A**) binding of purified full-length SRC to Ni-NTA agarose-bound His_6_-tagged PTP4A1 with Ni-NTA beads as control (*n* = 5) and (**B**) binding of GST-tagged SRC SH3SH2 to Ni-NTA agarose-bound His_6_-tagged PTP4A1 (*n* = 5). (**C**) SPR sensorgram (red) of oxidized PTP4A1 binding to immobilized His_6_-tagged SRC SH3SH2 with fit to a 1:1 kinetic model (black) and residuals plot (Δ). Two-tailed paired *t* test in **A** and **B**. ***P < 0.001.

We next sought to unravel the structural features of the complex between oxo-PTP4A1 and SRC. To this end, we developed robust protocols that enabled NMR data to be collected on fully oxidized or reduced PTP4A1 (i.e., no duplication of cross peaks is visible in their 2D [^1^H,^15^N] HSQC spectra) and determined their sequence-specific backbone assignments (Supplemental Figure 5). No differences in secondary structure between oxidized and reduced PTP4A1 were detected as assayed using chemical shift indexing. Reduced PTP4A1 displayed fewer visible NH cross peaks (81.6% versus 94.1% completeness), with missing residues clustered around the active site P-loop (that includes the catalytic Cys^104^) and helices α4 and α6 that flank the catalytically important WPD-loop, most likely due to intermediate exchange dynamics, indicating that reduced and oxidized PTP4A1 exhibited distinct behaviors in solution around the active site. To examine the involvement of different SRC domains amenable for a detailed analysis by NMR in the interaction, we then focused on the ability of covalently or noncovalently immobilized PTP4A1 to precipitate full-length SRC or its isolated SH3SH2, SH3, or SH2 domains. PTP4A1 efficiently precipitated full-length SRC, SRC SH3SH2, and SRC SH2 (Figure 3, A and B, and Supplemental Figure 6) but not SRC SH3 (data not shown). To confirm these findings and quantify the affinity between SRC and PTP4A1, we performed surface plasmon resonance (SPR) spectroscopy (21) using His_6_-tagged SRC SH3SH2 with in vitro oxidized or reduced PTP4A1. No robust signal was detected for reduced PTP4A1, while the interaction of SRC SH3SH2 with oxidized PTP4A1 resulted in a fast on/off kinetic profile with *K_D_* = 1.5 ± 0.2 μM (Figure 3C). Together, these data demonstrated that SRC SH3SH2 nearly exclusively bound oxidized PTP4A1 in vitro and justified the use of SRC SH3SH2 for further characterization by NMR spectroscopy.

**Figure 4:**
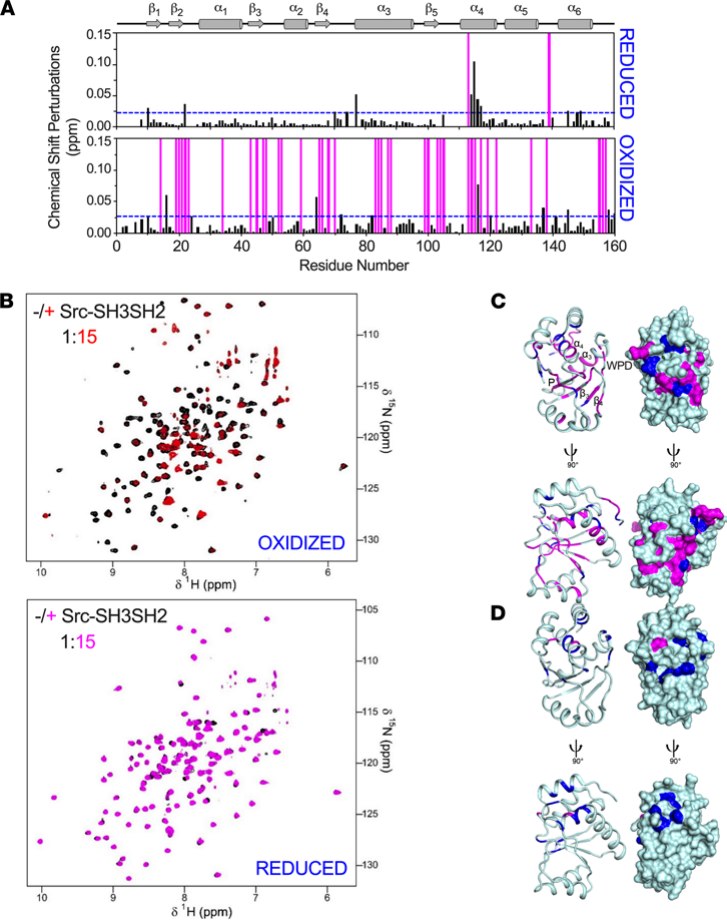
Oxidation-induced conformational changes in PTP4A1 P- and WPD-loops promote interaction with SRC. (**A**) Two-dimensional [^1^H,^15^N] TROSY spectra of oxidized and reduced PTP4A1 in the absence (black) or presence (red, magenta) of excess SRC SH3SH2. Blue dotted lines indicate 1σ levels, and residues that are line broadened beyond detection are indicated by magenta bars. (**B**) Diagram of CSPs of oxidized (top) and reduced (bottom) PTP4A1 upon binding of SRC SH3SH2. (**C** and **D**) Secondary structure elements are shown for reference. Residues showing chemical shift changes (blue; greater than 1σ) and intensity changes (magenta) for (**C**) oxidized and (**D**) reduced PTP4A1 were mapped onto the structures of oxidized (PDB ID 1RXD) and reduced (PDB ID 1XM2) PTP4A1 in ribbon and solvent-accessible surface representation. The key interacting regions of PTP4A1 are labeled.

To define the interaction between oxidized or reduced PTP4A1 and SRC SH3SH2 at atomic resolution, we recorded 2D [^1^H,^15^N] transverse relaxation-optimized spectroscopy (TROSY) spectra of (^15^N)-labeled PTP4A1 in the presence of increasing amounts of unlabeled SRC SH3SH2 (Figure 4A and Supplemental Figure 7), monitoring the resulting chemical shift perturbations (CSPs) and peak intensity changes to identify the SRC SH3SH2 binding interface on PTP4A1. For both reduced and oxidized PTP4A1, an interaction with SRC SH3SH2 was detectable. Titration of reduced PTP4A1 with SRC SH3SH2 resulted in only small CSPs, consistent with a μM-mM binding affinity. In contrast, titration of oxidized PTP4A1 with SRC SH3SH2 resulted in additional CSPs accompanied by significant changes in peak intensities, all hallmarks of a stronger association (Figure 4B), in agreement with the low μM *K_D_* measured by SPR. Furthermore, the majority of the CSPs and disappearing peaks in oxidized PTP4A1 belonged to residues around the P- and the WPD-loops, as well as adjacent residues in α4 and α6 (Figure 4, C and D). Critically, these are the same regions that were shown to exhibit changes in reduced versus oxidized PTP4A1. Thus, enhanced binding of SRC SH3SH2 correlated well with conformational and/or dynamic changes that occurred in the P- and WPD-loops upon PTP4A1 oxidation.

### oxo-PTP4A1 interacts with a continuous surface spanning the SRC SH3 and SH2 domains.

**Figure 5:**
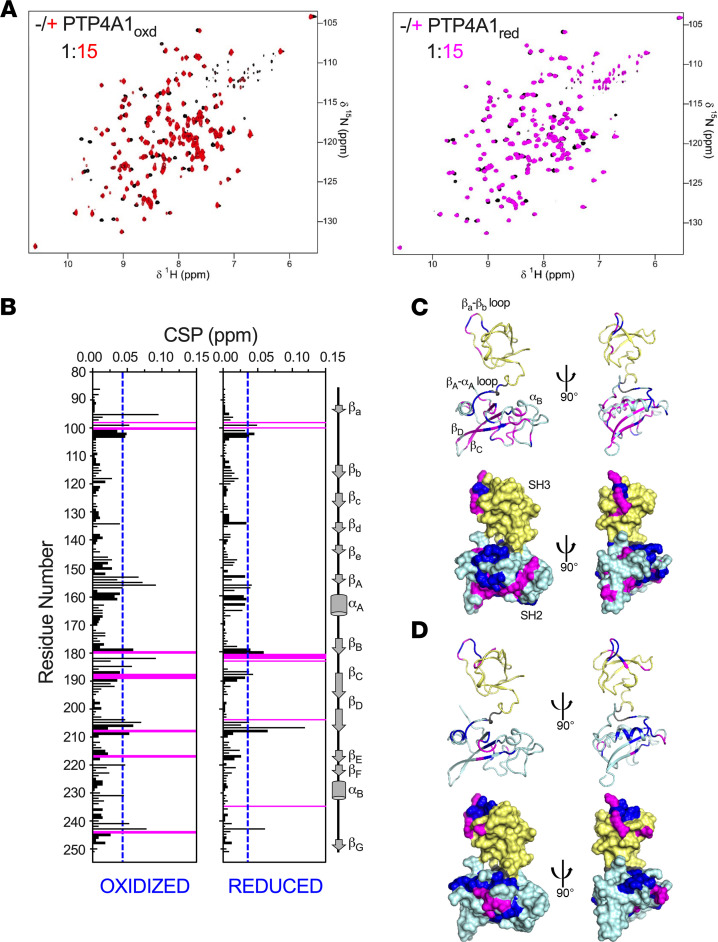
SRC binds PTP4A1 through its SH3 and SH2 domains. (**A**) Two-dimensional [^1^H,^15^N] TROSY spectra of SRC SH3SH2 in the absence (black) or presence of excess oxidized (red) and reduced (magenta) PTP4A1. (**B**) Diagram of CSP of SRC SH3SH2 upon binding of oxidized (left) and reduced (right) PTP4A1 as in Figure 4A and mapping of residues showing chemical shift changes (blue) and reduced intensity (magenta; I/I_0_ < 0.7) onto the structure of SRC SH3SH2 (PDB ID 2SRC) for (**C**) oxidized and (**D**) reduced PTP4A1. The key interacting regions of SRC SH2SH3 are labeled.

We next performed reverse titration experiments in which (^15^N)-labeled SRC SH3SH2 was titrated with increasing concentrations of either oxidized or reduced PTP4A1. First, we determined the sequence-specific backbone assignments for SRC SH3SH2 (96.4% completeness), and by comparing the individual 2D [^1^H,^15^N] HSQC spectra of the SRC SH3 and SH2 domains with that of SRC SH3SH2, we showed that the SH3 and SH2 domains behaved as independent entities in solution (data not shown). We next recorded 2D [^1^H,^15^N] TROSY spectra of (^15^N)-labeled SRC SH3SH2 in the presence of increasing amounts of unlabeled oxidized or reduced PTP4A1 (Figure 5A and Supplemental Figure 8). The addition of reduced PTP4A1 led to small CSPs. In contrast, addition of oxidized PTP4A1 led to more and larger CSPs and significant changes in peak intensities (Figure 5B and Supplemental Figure 8). Mapping of these CSPs and intensity changes onto the SRC SH3SH2 structure showed that PTP4A1 interacted with both the SH3 and SH2 domains via a largely continuous surface that included the SH3 β_A_-β_B_ loop (also referred to as RT-loop) and the SH2 domain β_A_-α_A_, β_B_-β_C_, β_C_-β_D_, and α_B_-β_G_ loops (Figure 5, C and D).

### oxo-PTP4A1 selectively forms a complex with open active SRC.

**Figure 6:**
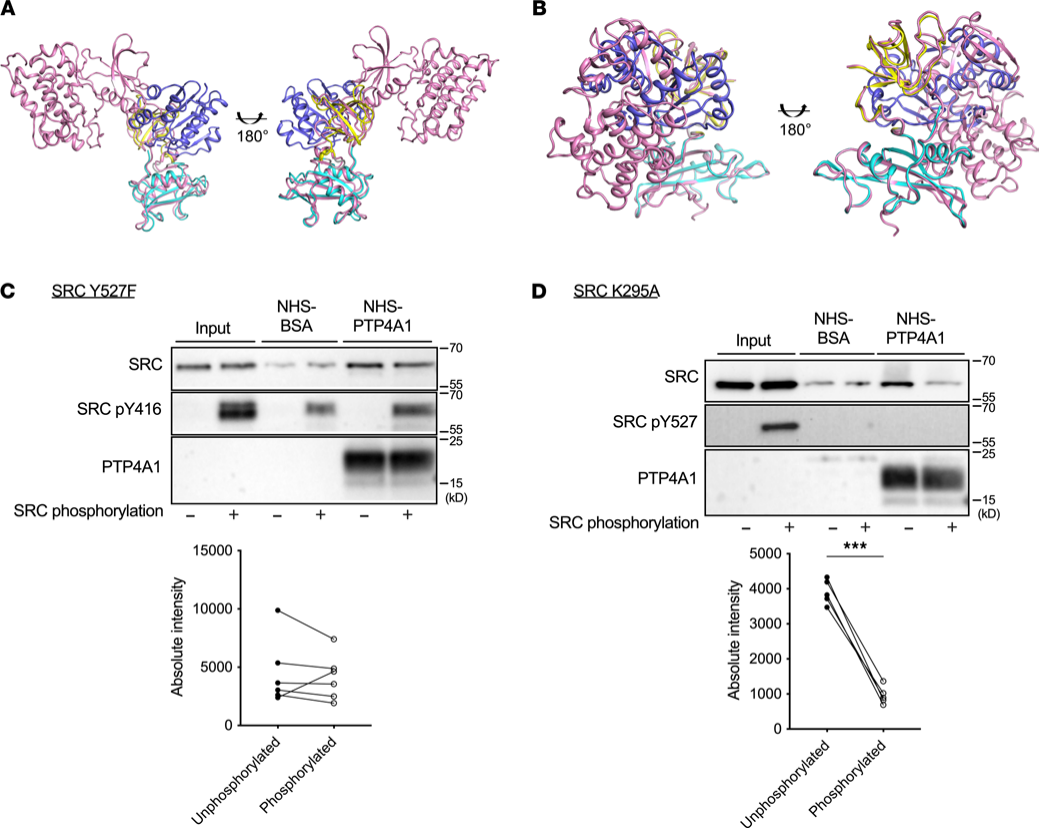
oxo-PTP4A1 selectively interacts with open SRC. (**A** and **B**) Structural superposition of (**A**) open (PDB ID 1Y57) and (**B**) closed (PDB ID 2SRC) structures of SRC (pink) with the NMR-based docking model of oxidized PTP4A1 (blue) with SRC SH3SH2 (SH3: yellow, SH2: cyan). (**C** and **D**) Representative Western blotting (top) with quantification (bottom) (*n* = 5) of (**C**) binding of purified autophosphorylated or unphosphorylated SRC^Y527F^ to covalently immobilized PTP4A1 and (**D**) binding of purified CSK-phosphorylated or unphosphorylated SRC^K295A^ to covalently immobilized PTP4A1. Two-tailed paired *t* test in **C** and **D**. ***P < 0.001.

We next created an SRC SH3SH2-oxo-PTP4A1 complex model using HADDOCK and our NMR data (Supplemental Figure 9). As expected, the model showed that oxidized PTP4A1 helices α3 and α4 and β-strands β3 and β4 along with the P-loop and WPD loop made extensive contacts with helix αB and β-strands βC and βD along with the βA-αA loop and βA-βB loop of SRC SH3SH2. The complex interface buried 1010 Å^2^, consistent with the observed *K_D_* of approximately 1.5 μM. SRC is regulated by phosphorylation events on Y^416^ and Y^527^. Phosphorylation of Y^527^ by CSK stabilizes SRC in a closed and inactive conformation (11, 22). We noticed that our NMR-based model was compatible with binding of PTP4A1 to open (22) but not to closed (11) SRC because of steric clashes (Figure 6, A and B). We therefore compared the binding of PTP4A1 to SRC selectively phosphorylated on either Y^416^ (active) or Y^527^ (inactive) using 2 SRC variants that only allow phosphorylation on a single site: (1) SRC^Y527F^, which cannot be phosphorylated at Y^527^, and (2) the kinase-dead mutant SRC^K295A^ (23), which undergoes specific phosphorylation of Y^527^ by CSK but is unable to autophosphorylate at Y^416^ (see Supplemental Figure 10). In coprecipitation assays, PTP4A1 bound to unphosphorylated and Y^416^-phosphorylated SRC to similar extents (Figure 6C). In contrast, PTP4A1 was unable to bind SRC^K295A^ phosphorylated on Y^527^ (Figure 6D). Selective binding of oxo-PTP4A1 to open SRC is consistent with its SRC-promoting function and suggests that oxo-PTP4A1 might stabilize SRC in its open/active conformation.

### The complex between PTP4A1 and SRC contributes to ROS-induced amplification of TGF-β signaling.

**Figure 7:**
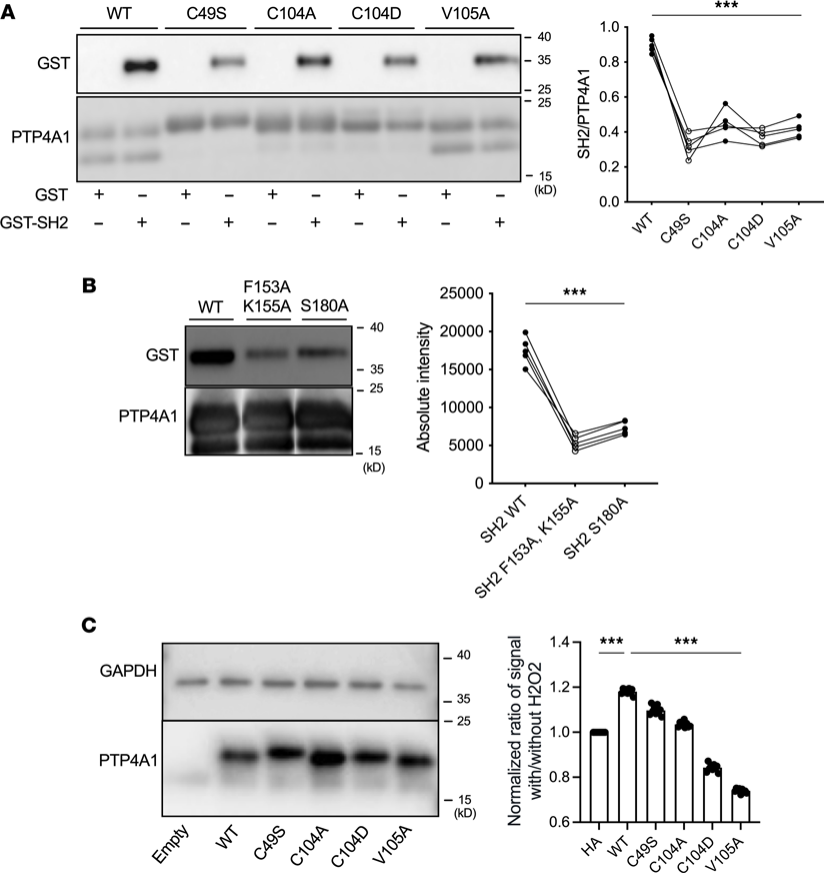
Mutational analysis of PTP4A1-SRC interaction and its effect on TGF-β signaling. (**A** and **B**) Representative Western blotting (top) with quantification (bottom) (*n* = 5) of (**A**) GST coprecipitation assay of binding between PTP4A1 mutants C49S, C104A, C104D, and V105A with GST-tagged SRC SH2 normalized to PTP4A1 mutant loaded on the beads, and (**B**) binding between F153A/K155A and S180A mutants of GST-tagged SRC SH2 to PTP4A1. (**C**) Representative Western blotting of lysates from TGF-β–stimulated HEK293T cells transfected with WT or mutant PTP4A1 (left) with quantification of luciferase luminescence from SMAD reporter assay (right). The signal from the cells treated with H_2_O_2_ was normalized to untreated ones. Data shown are mean ± SEM. *n* = 9. One-way ANOVA in **A** and **B**, 1-way ANOVA with multiple-comparison test in **C**. ***P < 0.001.

We next assessed whether mutation of key residues in the PTP4A1-SRC interface affects the interaction of PTP4A1 with SRC and the effect of oxidative stress on TGF-β signaling. PTP4A1^C49S^, PTP4A1^C104A^, and PTP4A1^C104D^, which cannot form the disulfide bond in oxidized PTP4A1, as well as PTP4A1^V105A^, purified readily and exhibited decreased affinity toward the SRC SH2 domain (Figure 7A). Also consistent with the data shown in Figure 5, C and D, SRC SH2^F153A,K155A^ and SRC SH2^S180A^ were observed to have weaker interaction with PTP4A1 (Figure 7B). We thus probed the ability of these PTP4A1 mutants to modulate TGF-β signaling in the context of oxidative stress using a SMAD3-dependent reporter assay. Figure 7C shows that while WT PTP4A1 enhanced TGF-β signaling under oxidative conditions, this effect was reduced or even reversed by the PTP4A1^C49S^, PTP4A1^C104A^, PTP4A1^C104D^, and PTP4A1^V105A^ mutations expressed at the same level (Figure 7C), demonstrating that the PTP4A1-SRC complex promoted SMAD-mediated TGF-β signaling in the context of oxidative stress.

### Inducible global deletion of PTP4A1 prevents and halts progression of TGF-β–induced fibrosis.

**Figure 8:**
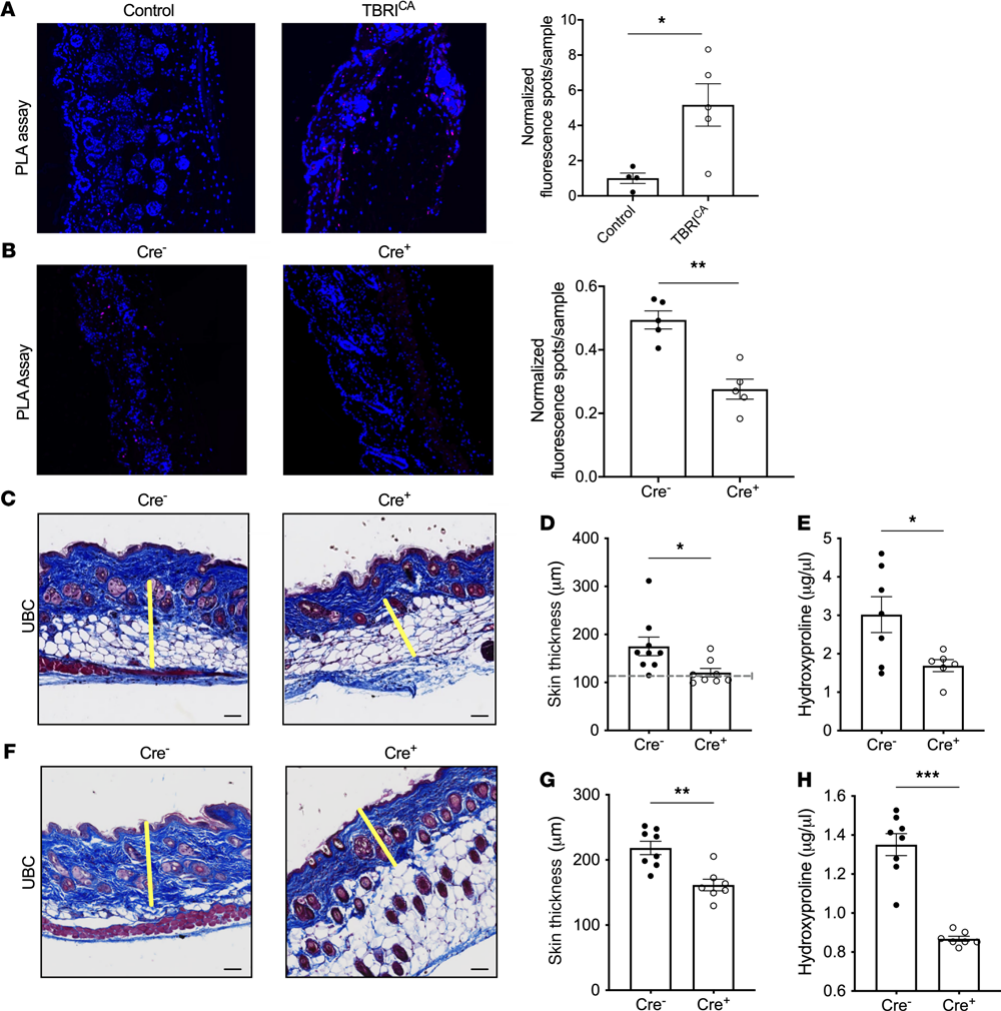
Global inducible deletion of PTP4A1 prevents and reduces progression of fibrosis. (**A**) Representative PTP4A1-SRC PLA signal in skin specimens from TBRI^CA^-expressing or control mice (left) with quantification (right), *n* ≥ 4. (**B**) Representative PLA signal in skin from *Col1a1*-Cre mice treated with tamoxifen before induction of fibrosis (left) with quantification (right), *n* = 5. (**C** and **F**) Representative Masson’s trichrome staining of skin from *Ubc*-Cre mice treated with tamoxifen before (**C**) or after (**F**) induction of fibrosis. Yellow lines show representative quantification of dermis thickness. Twenty or more measurements were taken across each section. (**D** and **G**) Quantification of skin thickness in specimens from **C** and **F**, respectively. (**D**) Dotted line shows average normal dermal thickness. (**E** and **H**) Quantification of hydroxyproline on specimens from **C** and **F**, respectively. (**C**–**E**) *n* ≥ 5, (**F**–**H**) *n* ≥ 7. (**A** and **B**) Blue = DAPI, magenta = PLA. Images were captured with 20× original magnification. All data are mean ± SEM. Data in **A** and **B** are shown normalized to the control averages. Two-tailed Mann-Whitney test in **A**, **B**, **D**, and **H**; 2-tailed Welch’s *t* test in **E** and **G**. *P < 0.05, **P < 0.01, ***P < 0.001.

PTP oxidation and degradation have recently emerged as a potential druggable angle of PTP physiology (24, 25). To evaluate the potential of PTP4A1 as a drug target for SSc, we assessed whether global inducible deletion of PTP4A1 could ameliorate disease in a model of SSc dependent on the constitutively active TGF-β receptor type I (TBRI^CA^) and oxidative stress (20, 26). PLA assays performed on skin from healthy mice versus fibrotic TBRI^CA^ mice showed a strong induction of the PTP4A1-SRC complex in dermal fibroblasts from fibrotic mice (Figure 8A and Supplemental Figure 11). Inducible fibroblast-selective *Ptp4a1* KO driven by inducible *Col1a1*-Cre driver prevented skin fibrosis in TBRI^CA^ mice as measured by skin thickness, hydroxyproline levels, and reduced expression of the 2 fibrosis markers *Acta2* and *Col1a1*. Importantly, it also removed the elevated PLA signal observed in WT fibrotic skin, confirming the cell and target specificity of our PLA results (Figure 8B and Supplemental Figure 11). We thus assessed whether fibrosis could be prevented or halted in its progression in the TBRI^CA^ model by global induced *Ptp4a1* KO. As shown in Figure 8, C–H, and Supplemental Figure 12, *Ubc*-Cre–driven induction of global *Ptp4a1* KO both prevented disease and significantly limited progression of established disease in this model without evident lethality or clinical signs of toxicity.

## Discussion

TGF-β has long been known as a key regulator of fibrotic phenotype and has been suggested as a target for the treatment of SSc and other fibrotic diseases. However, because of its involvement in a wide range of cellular processes, indiscriminate attenuation of TGF-β signaling for therapeutic purposes is unlikely to succeed, absent a thorough understanding of its downstream cellular events (27). Here, we present strong evidence of a direct SRC-PTP4A1 interaction in cells that provides a molecular link between TGF-β stimulation, oxidative stress, SRC activation, and fibrosis in SSc. We showed that SRC kinase preferentially interacted with PTP4A1 in its open and active state, which could directly promote SRC activity, and moreover reduce levels of inactive pY^527^-SRC by keeping the newly phosphorylated C-terminal tail exposed to the action of phosphatases, consistent with our previous observation of increased Y^527^ phosphorylation in PTP4A1 knockdown fibroblasts (15). Intriguingly, PTP4A1 selectively interacted with SRC in its oxidized state. Oxidation of the active site Cys of PTPs by ROS via formation of sulfenic acid — which leads in a subset of PTPs, such as PTP4A1, to disulfide bridge formation — is a well-known mechanism for physiological and pathological downregulation of their activity (6) and has been proposed to play a role in the pathogenesis of SSc fibrosis by promoting growth factor signaling (7). However, here we unexpectedly showed that PTP oxidation involving the catalytic Cys can also promote its function depending on signaling platforms and interacting partners. The catalysis-independent role of PTP4A1 in fibrosis is consistent with the current view that PTP4A enzymes function, at least in part, as pseudophosphatases (28).

To further support our model, we have elucidated the molecular details of the complex between oxidized PTP4A1 and SRC. The P- and the WPD-loops, as well as adjacent secondary structural elements of PTP4A1, exhibited changes upon oxidation, which allowed for a change in their ability to mediate protein-protein interactions. The involvement of residues around the active site is consistent with the different affinity for the oxidized versus reduced state. It has been previously shown that oxidation of PTP4A proteins reduces their affinity for the CNNM transporters (16, 17). Although the CNNM channels do not mediate the profibrotic effect of PTP4A1 in TGF-β signaling (15), the differential regulation of PTP4A1-CNNM versus PTP4A1-SRC complexes by oxidation suggests a mechanism for balancing the pool of oxidized PTP4A1 between different PTP4A1 interactors.

The role of oxidation in PTP4A1-SRC complex stability might also underlie the observed differences in intracellular association between SRC and PTP4A1 versus PTP4A2, despite the high sequence and 3D similarities between PTP4A enzymes. Although further work is needed to unravel the differential regulation of SRC by PTP4A1 versus PTP4A2 in fibroblasts, differences in the sensitivity of PTP4A1 versus PTP4A2 toward oxidative stress and the regulation of their oxidation state in cells are promising areas for further exploration.

Oxidative stress plays an important role in fibroblast dysfunction and mechanism of disease in SSc (29, 30). Production of ROS plays a role in signaling by TGF-β and PDGF (5, 7, 31–33), 2 key profibrotic factors in SSc, and higher levels of ROS have been reported in SSc fibroblasts and in tissues and fluids from patients with SSc (7, 34, 35). A link between ROS and SRC signaling in scleroderma fibroblasts was previously reported (36). Oxidation and inactivation of PTP1B has been proposed to play a role in the pathogenesis of SSc fibrosis by promoting growth factor signaling (7, 32). The striking differences in SRC binding by reduced versus oxidized PTP4A1 and the high specificity of our PLA assay therefore point to a strong induction of an oxo-PTP4A1-SRC complex in fibrotic skin from patients with SSc and mouse models of disease as well as in ex vivo cultured dermal fibroblasts subjected to TGF-β stimulation. Since a large fraction of PTP4A1 is kept in a reduced state in the resting NHDFs and other cell types (our unpublished data and ref. 37) and we observed consistent reduction of oxo-PTP4A1-SRC complex levels after attenuation of endogenous ROS production in SScDFs, we conclude that oxidative stress is at least in part responsible for oxo-PTP4A1-SRC complex formation in SScDFs and SSc fibrotic skin and the subsequent SRC-mediated promotion of TGF-β signaling. However, it should be noted that data obtained in SScDF lines — although supported in this study by similar PLA signals in mouse and SSc skin specimens — might not accurately recapitulate the behavior of SSc fibroblasts in vivo. Furthermore, it is currently not technically feasible to either directly demonstrate oxidation of PTP4A1 in tissue sections or preserve PTP4A1 oxidation state in sorted primary fibroblast populations. This limits our ability to assess the relevance of the abovementioned mechanism of action in the context of the emerging awareness of fibroblast heterogeneity in SSc pathogenesis.

PTP oxidation has recently emerged as a potential druggable angle of PTP physiology (3). Chemical promotion of catalytic Cys oxidation enabled potentially therapeutically relevant modulation of PTP1B activity in cells (24). Considering that inducible systemic *Ptp4a1* KO did not result in any clinically evident adverse effect in mice, the work described in this paper suggests that further validation of PTP4A1 as a selective drug target for SSc and potentially other fibrotic diseases might be warranted.

In conclusion, we propose an unusual mechanism of regulation of a PTP that is induced in SSc skin, whereby oxidation of PTP4A1 by ROS synergizes with induction of PTP4A1 expression by TGF-β signaling to promote the formation of the complex between PTP4A1 and SRC. This in turn renders fibroblasts more sensitive to stimulation by TGF-β and potentially other profibrotic factors. We propose this mechanism is active in SSc fibrosis where higher levels of ROS are driven by ischemia and multiple proinflammatory factors (4, 7) and could be targeted therapeutically for potentially novel and personalized antifibrotic approaches for SSc (24, 25).

## Methods

### Abs.

The mouse anti-PTP4A1/2 (clone 42) Ab was purchased from MilliporeSigma. The mouse anti-SRC (L4A1), normal rabbit IgG (2729S), rabbit anti-SRC (2108S), rabbit anti-pSRC (Y^416^, 2101), rabbit anti-pSRC (Y^527^, 2105), and rabbit anti-GST (2625S) Abs were purchased from Cell Signaling Technology.

### Study participants and cell lines.

All the NHDF and SScDF lines utilized in this study have been previously described and were utilized between passage 5 and 9 (15). NHDFs/SScDFs and skin biopsies were obtained from the UCSD Rheumatology Section (UCSD IRB 140485) and the UCSF Scleroderma Center (UCSF IRB 15-16463), respectively. NHDFs were also obtained through the NDRI tissue bank (Philadelphia, Pennsylvania, USA) and from the ATCC. All patients with SSc met the 2013 American College of Rheumatology/European League against Rheumatism criteria (38). The SSc cohorts included 12 White patients, 2 African American patients, and 1 Asian patient with diffuse SSc between 21 and 69 years old; the control cohort included 6 White healthy individuals between 25 and 41 years old (see Supplemental Table 1 for details). HEK293T cells were obtained from ATCC and were cultured in DMEM supplemented with 10% FBS with 10 mM HEPES pH 7.0 and penicillin and streptomycin.

### PLAs.

NHDFs or SScDFs were seeded on sterile coverslips in FBS-supplemented DMEM until 50% confluence, starved for 24 hours, and stimulated with 50 ng/mL TGF-β for 6 hours. For the NAC experiment, the cells were treated with 20 mM NAC or left in normal starvation media overnight after TGF-β stimulation. Cells were fixed with 4% PFA for 20 minutes and permeabilized with 0.2% Triton X-100 for 5 minutes. PLAs were performed according to the manufacturer’s protocol (MilliporeSigma) with 1:50 mouse anti-PTP4A1/2 and 1:100 rabbit anti-SRC Ab. Some samples were visualized with an Olympus FV10i confocal microscope and 60× 1.35 NA oil objective UPLSAP60xO using FluoView 2.1.1.7 software. Fluorescence was collected through 1.0× confocal aperture on bialkali photomultiplier tubes (550 V and 513 V, respectively) with 2 μs pixel dwell time and 2× line averaging in line-switching mode to minimize crosstalk. Next, 15–20 slices were taken for a minimum of 19 cells in each tested condition per cell line. Some samples were visualized using a Zeiss Axiovert Mariana 200M using a 40× 0.55 NA oil objective using SlideBook 6 software. Images were captured as a 3 × 3 montage with a minimum of 10 cells in each montage image. All samples were excited with 405 nm and 559 nm lasers, and fluorescence of Hoechst (420–460 nm) and PLA probe (570–670 nm) was collected. Maximum intensity projections were created in Fiji (39), and the number of PLA spots was evaluated with Find Maxima function.

Additionally, human and murine tissue sections were deparaffinized with xylene and were then incubated in 100%, 96%, 80%, and 70% ethanol. After incubation in aqua dest., sections were washed with PBS. The tissue was incubated with 1× trypsin/EDTA in a humid chamber for 10 minutes at 37°C. After digestion, the probes were washed with PBS and blocked in 10% normal horse serum for an hour. Afterward, the sections were stained as described above. The PLA signal was visualized with a Zeiss Axioscan Z1 using a 20× objective. Fiji software was used for signal quantification by counting the number of PLA spots per tissue. As negative control for all PLA experiments, primary Abs were individually or completely omitted from the experiment.

### Subcloning, mutagenesis, protein expression, and purification.

For coprecipitation assays, a codon-optimized sequence encoding human PTP4A1 (residues 1–160) was subcloned into the NdeI/BamHI site of pET28a (Novagen) and expressed in *E*. *coli* BL21 (DE3) as an N-terminal His_6_ fusion. Bacterial cultures were grown at 37°C and induced with isopropyl-1-thio-β-d-galactopyranoside (IPTG) at room temperature overnight. After recovery from the soluble fraction of the cell lysate, PTP4A1 was purified by Ni-NTA affinity chromatography (Qiagen) and cation exchange chromatography (Bio-Rad, 158-0040) at pH 7.3. When the His_6_-tag was not needed, thrombin was used to cleave it followed by size-exclusion chromatography (ENrich SEC 650, Bio-Rad). A codon-optimized sequence encoding full-length human SRC was subcloned in the BamHI/EcoRI site of pGEX-4T-1, coexpressed with *Yersinia* YopH (40) phosphatase as an N-terminal GST fusion in *E*. *coli* BL21 (DE3), and purified from the soluble fraction of the lysate by glutathione affinity chromatography. The GST tag was removed by digestion with thrombin, a second step of Glutathione Agarose Affinity Chromatography (MilliporeSigma, G4510) and a size-exclusion chromatography step. For SRC SH3 (residues 81–142), SH2 (residues 148–245), and SH3SH2 (residues 81–248), the corresponding regions were PCR amplified, cloned, expressed, and purified following the same procedures as SRC except that no coexpression with YopH phosphatase was performed and the GST tag was not removed. All PTP4A1, SRC, and SRC SH2 mutants were generated by standard site-directed mutagenesis techniques and purified as described above. CSK (residues 1–443) was subcloned into the NcoI/XhoI site of pET28a (Novagen) and expressed as a C-terminal His_6_ fusion. The expression was induced with IPTG at 18°C for 16 hours, and the protein was purified by Ni-NTA affinity chromatography (Qiagen) and size-exclusion chromatography (ENrich SEC 650, Bio-Rad). All the plasmid constructs and mutations were confirmed by DNA sequencing. All primers used were synthesized by Integrated DNA Technologies.

### In vitro coprecipitation assays.

To prepare Ni-NTA agarose-bound PTP4A1 beads for coprecipitation assays, 0.1 mg His_6_-tagged PTP4A1 in 1 mL of 150 mM NaCl and 20 mM Tris pH 7.3 was mixed with 100 μL Ni-NTA agarose beads (Qiagen) at 4°C overnight. The beads were then washed and resuspended in 1 mL buffer. To prepare agarose beads covalently coupled to PTP4A1, 0.5 mg PTP4A1 in 1 mL PBS was mixed with 75 mg dry *N*-hydroxysuccinimide–activated (NHS-activated) agarose beads (Thermo Fisher Scientific) at 4°C overnight. After several washes, unreacted NHS groups were quenched with 1 mL 1 M ethanolamine, pH 8.2, at room temperature for 20 minutes. The beads were washed again and resuspended in 1 mL PBS. For the coprecipitation assays, 0.5 μg SRC in 1 mL binding buffer (60 mM HEPES pH 7.4, 5 mM MgCl_2_, 5 mM MnCl_2_, 1% BSA) was combined with 20 μL bead suspension at 4°C for 2 hours. The beads were washed 3 times with the same buffer (containing additional 50 mM imidazole) and assessed by Western blotting. To prepare pY^416^ SRC, 0.05 μg/μL SRC^Y527F^ were incubated at 30°C for 5 minutes in a buffer containing 300 mM NaCl, 20 mM Tris pH 8.0, 10 mM MgCl_2_, 10 mM DTT, and 1 mM ATP. To prepare pY^527^ SRC, 0.05 μg/μL SRC^K295A^ and 0.025 μg/μL CSK were incubated in the same buffer and conditions as above. Phosphorylation was verified by Phos-tag SDS-PAGE and Western blotting.

Autophosphorylation led to phosphorylation of SRC^Y527F^ on a single site starting from 15 minutes. Significant phosphorylation on other minor sites appeared at longer reaction times and became preponderant at 16 hours. CSK phosphorylation led to selective Y^527^ phosphorylation of SRC^K295A^ and was largely complete at 15 minutes (Supplemental Figure 10). To test the effect of the oxidation state of PTP4A1, the PTP4A1-bound beads were incubated in 1 mL binding buffer with either 0.5 mM H_2_O_2_ or 20 mM DTT for 1 hour prior to the coprecipitation assay. To assess the binding of different SRC domains, 86 nmol of GST-tagged SH2 or SH3 or 17.2 nmol GST-tagged SH3SH2 were used for the assay.

### Co-IP.

HEK293T cells were seeded in 6-well plates, cultured to around 90% confluence, and transfected in Opti-MEM reduced serum medium (Thermo Fisher Scientific) for 24 hours with 2 μg/well pcDNA4 plasmid encoding HA-tagged PTP4A1 preincubated with 6 μg polyethylenimine (PEI). To oxidize PTP4A1, the cells were incubated with 1 mM H_2_O_2_ directly added to the medium for 30 minutes at 37°C. The cells were lysed in TNE buffer containing 1% Triton X-100 40 mM *N*-ethylmaleimide (Thermo Fisher Scientific) and a complete EDTA-free protease inhibitor cocktail (Roche), centrifuged to remove insoluble debris and precleaned by incubation with Protein G Sepharose beads (Cytiva). Rabbit anti-SRC Ab was then added to the cell lysate at 1:100 dilution for 12 hours before incubation with Protein G Sepharose beads for 2 hours. The beads were then washed and resuspended in Laemmli buffer for SDS-PAGE and immunoblotting.

### SMAD reporter assay.

HEK293T cells were seeded in 96-well plates, cultured to around 90% confluence, and transfected in Opti-MEM reduced serum medium (Thermo Fisher Scientific) for 24 hours with 1 μL SMAD reporter (Qiagen), 50 ng pcDNA4 plasmid encoding HA-tagged WT or mutant PTP4A1 per well preincubated with 2.25 μg PEI. For oxidizing condition, the cells were treated with 100 μM H_2_O_2_ for 30 minutes before stimulation with 20 ng/mL TGF-β for 6 hours. After washing with PBS, the cells were lysed and dual luciferase assay (Promega) was performed according to the manufacturer’s protocol. The luminescence was measured with a TECAN Infinite F PLEX plate reader.

### Protein production for SPR and structural studies.

The coding sequences of human PTP4A1 (residues 1–160) and SRC SH3SH2 (residues 84–251) were subcloned into pRP1B vector containing an N-terminal His_6_-tag and a tobacco etch virus (TEV) cleavage site. *E*. *coli* strain BL21-Codon-Plus (DE3) RIL (Agilent) cells were transformed with PTP4A1 and SRC SH3SH2 expression vectors. Freshly transformed cells were grown at 37°C to an OD_600_ of 0.8–1 and induced with 1 mM IPTG overnight (18 to 20 hours) at 18°C. Cells were harvested by centrifugation (6000*g*, 15 minutes, 4°C) and stored at –80°C until further purification. Expression of uniformly ^15^N- or [^15^N,^13^C]-labeled proteins was achieved by growing cells in M9 minimal media containing 1 g/L ^15^NH_4_Cl and 4g/L [^13^C]-d-glucose (CIL) or d-glucose as the sole nitrogen and carbon sources, respectively.

PTP4A1 or SRC SH3SH2 cell pellets were resuspended in ice-cold lysis buffer (50 mM Tris pH 8.0, 500 mM NaCl, 5 mM imidazole, 0.1% Triton X-100, and an EDTA-free protease inhibitor tablet from Roche) and lysed by high-pressure homogenization (Avestin Emulsiflex C3). Lysate was clarified by centrifugation at 42,000*g* for 45 minutes at 4°C. The supernatant was loaded onto Ni^+2^- NTA (nitrilotriacetic acid) beads pre-equilibrated with 50 mM Tris pH 8.0, 500 mM NaCl, and 5 mM imidazole and eluted using 50 mM Tris pH 8.0, 500 mM NaCl, and 500 mM imidazole. Eluted protein was dialyzed with TEV protease at 4°C against 50 mM Tris, pH 8.0, and 500 mM NaCl to cleave the His_6_-tag. The cleaved protein was incubated with Ni^+2^-NTA beads to remove TEV protease and cleaved His_6_-tag. The flow-through was concentrated and further purified using size-exclusion chromatography (Superdex 75 26/60; Cytiva) equilibrated in 10 mM HEPES pH 7.4, and 150 mM NaCl or 10 mM HEPES pH 7.4, 150 mM NaCl, and 10 mM DTT.

### Oxidation and reduction of PTP4A1 for NMR and SPR studies.

The preparation of uniformly reduced PTP4A1 was accomplished by incubating PTP4A1 with 10 mM DTT at 40°C for 2 hours. The preparation of uniformly oxidized PTP4A1 was accomplished by incubating with 40 mM oxidized glutathione at 25°C for 16 hours. The oxidation state of PTP4A1 was monitored by measuring the melting temperature using a Tycho NT.6 (Nanotemper) using standard capillaries (10 μL) using a 30°C/min ramp (from 35°C to 95°C) and evaluated using Tycho NT.6 software version 1.1.5.668. The oxidized, reduced, and mixture of PTP4A1 species showed melting temperatures of 71°C, 77°C, and 74°C, respectively.

### NMR spectroscopy.

All NMR experiments were acquired at 313 K on Bruker Avance NEO 600 MHz or 800 MHz (^1^H Larmor frequency) NMR spectrometer equipped with a TCI-active HCN-active z-gradient cryoprobe. The interaction between ^15^N-labeled reduced or oxidized PTP4A1/SRC SH3SH2 with unlabeled SRC SH3SH2/reduced or oxidized PTP4A1, respectively, was studied by direct comparison of 2D [^1^H,^15^N] TROSY spectra of free (reduced or oxidized PTP4A1/SRC SH3SH2) and bound (SRC SH3SH2/reduced or oxidized PTP4A1, respectively). The titration was performed at increasing molar ratios (1, 2, 5, 10, and 15) of unlabeled reduced or oxidized PTP4A1/SRC SH3SH2 to monitor the CSP and intensity changes. The final concentration of free reduced or oxidized PTP4A1/SRC SH3SH2 was 0.1 mM in 10 mM HEPES pH 7.4, and 150 mM NaCl or 10 mM HEPES pH 7.4, 150 mM NaCl, and 10 mM DTT containing 10% D_2_O. The spectra were processed using Topspin 4.0.6 (Bruker) and analyzed using NMRFAM-Sparky. Backbone amide chemical shift deviations were calculated using the formula: Δδ_av_ = sqrt (0.5[(δ_HN,bound_–δ_HN,free_)^2^ + 0.04(δ_N,bound_–δ_N_,_free_)^2^]). The sequence-specific backbone resonance assignment of reduced/oxidized PTP4A1 and SRC SH3SH2 (recorded at 600 MHz ^1^H Larmor frequency) was achieved by recording the following spectra at 298 K or 313 K at a final protein concentration of 0.7–1 mM: 2D [^1^H,^15^N] HSQC, 3D HNCACB, 3D CBCA(CO)NH, 3D HNCA, 3D HN(CO)CA, 3D (H)CC(CO)NH, and 3D HNCO. The buffers used for these experiments were 20 mM sodium phosphate pH 6.5, 150 mM NaCl, and 10% D_2_O (for oxidized PTP4A1 and SRC SH3SH2) and 10 mM HEPES pH 7.4, 150 mM NaCl, 10 mM DTT, and 10% D_2_O (for reduced PTP4A1). All spectra were processed using Topspin 4.0.6 (Bruker), and chemical shift assignments were achieved using the software CARA (http://cara.nmr.ch).

### SPR.

SPR measurements were performed using a 4-channel Reichert 4SPR instrument fitted with an autosampler and degassing pump. SPR running buffer containing 20 mM Tris pH 8.0, 250 mM NaCl, 0.05% Tween 20 were prepared, sterile filtered, and degassed in glassware prior to each experiment. Both the sample and syringe pump reservoirs were primed with running buffer prior to each experiment. Gold sensor chips modified with Ni-NTA-functionalized dextran (NiD200M; Xantec) were installed and equilibrated under flow conditions (100 μL/min) for 60 minutes or more at 25°C. Experiments were conducted at 25°C with a 5 Hz sampling rate and were initiated by injecting 90 μL of His_6_-SRC SH3SH2 (20 nM) diluted in SPR running buffer onto channels 1 and 2 for 90 seconds at 50 μL/min, which resulted in between 100 and 110 μRIU surface loading, with channels 3 and 4 being used as reference surfaces. Single 60 μL injections of purified oxidized PTP4A1 diluted into SPR running buffer were applied for 60 seconds at 50 μL/min followed by a 120-second dissociation step. Technical replicates were obtained by utilizing 2 channels per chip coupled with stripping of the sensor chip with 350 mM EDTA pH 8.0, reconditioning the surface with 10 mM NaOH to remove nonspecifically bound PTP4A1, charging the surface with 40 mM NiSO_4_, and reloading fresh His_6_-SRC SH3SH2. All replicates were generated with freshly diluted His_6_-SRC SH3SH2 and PTP4A1. Single injection kinetic parameters were determined by curve-fitting using TraceDrawer software (Ridgeview Instruments AB) fit with a 1-to-1 model.

### Docking model.

The following is the NMR-based model for the SRC SH3SH2: oxidized PTP4A1 complex was generated using HADDOCK (41) and ambiguous NMR-derived restraints as inputs. The SRC SH3SH2 coordinates were derived from c-Src^83-533^ (comprising the SH3, SH2, and catalytic domains, PDB ID 2SRC) (11) and oxidized PTP4A1 (PDB ID 1RXD) (42) were used as inputs. Active residues were defined as those that experienced a CSP greater than or equal to the mean plus 1σ or were broadened beyond detectability and had high solvent accessibility in the unbound protein structure as calculated using GetArea (43). Active SRC SH3SH2 residues were 97, 147, 152, 154, 179, 182, 205, 206, 228, and 233; active PTP4A1 residues were 22, 154, 155, and 156. Passive residues were defined as those that had CSPs or were broadened beyond detectability and had a low solvent accessibility. Passive SRC SH3SH2 residues were 92, 95, 96, 99, 100, 150, 153, 155, 161, 167, 170, 171, 172, 173, 174, 175, 176, 177, 185, 186, 188, 189, 190, 191, 192, 196, 198, 200, 201, 202, 203, 204, 214, 217, 218, 231, 235, 237, 238, 240, 241, 243, and 245; passive oxidized PTP4A1 residues were 13, 18, 19, 20, 21, 33, 42, 44, 46, 47, 51, 52, 58, 64, 65, 67, 69, 82, 83, 84, 86, 87, 98, 99, 102, 103, 104, 112, 113, 114, 116, 118, 121, 132, and 137. The residues that showed CSPs or were broadened beyond detectability were the only experimental restraint type used in the HADDOCK calculation, and default settings were used for all docking steps. All the residue numbers correspond to the numbering of residues as in the PDB files.

### Intracellular ROS assay.

SScDFs were seeded on fibronectin-coated wells of a 96-well plate and grown to 90% confluence before overnight serum starvation. Cells were then stimulated with 50 ng/mL TGF-β for 6 hours or left unstimulated, and stimulated cells were then incubated overnight in 20 mM NAC or normal starvation media. SScDFs were subsequently stained with 20 μM DCFDA (Abcam) according to the manufacturer’s instructions, and intracellular ROS levels were quantified by measuring fluorescence on a Tecan Infinite 200 Pro microplate reader with excitation/emission set to 485 nm/535 nm.

### Mouse studies.

All mice were housed in the UCSD-ACTRI vivarium under specific pathogen–free conditions. The studies in animals were conducted in accordance with protocols approved by the IACUC of UCSD (protocol Bottini-S16098). Eight-week-old female C57BL/6 mice were obtained from The Jackson Laboratory. *Ptp4a1^fl/fl^* and *Ptp4a1^fl/fl^*-*Col1a1* mice on C57BL/6 background have been previously described, and the original strains were obtained from Klaus H. Kaestner (University of Pennsylvania, Philadelphia, Pennsylvania, USA) and The Jackson Laboratory (strain 016241), respectively (15). *Ptp4a1^fl/fl^* were mated with B6.Cg-Tg(Ubc-Cre/ERT2)1Crm/J mice purchased from The Jackson Laboratory. To demonstrate that *Ptp4a1* deletion could prevent fibrosis in the TBRI^CA^ model, Cre expression was induced postnatally in 7-week-old female *PTP4A1^fl/fl^-Ubc-Cre* mice through i.p. or oral gavage administration of 2 mg tamoxifen dissolved in 100 μL corn oil for 5 consecutive days (15). Then, skin fibrosis was induced by injecting the mice with 6.67 × 10^7^ PFU of replication-deficient type 5 adenoviruses encoding TBRI^CA^ (Vector Biolabs) into a defined area of 1–2 cm^2^ at the upper back every 2 weeks for 4 times total according to a published protocol (44). To test whether *Ptp4a1* deletion suppresses the progression of fibrosis in the TBRI^CA^ model, 7-week-old male *PTP4A1^fl/fl^-Ubc-Cre* mice were first injected with the adenoviruses for 3 times total, and the same tamoxifen treatment was started 1 week later.

### Mouse tissue histology.

Mouse skin specimens were obtained from the injection area using disposable biopsy punchers, fixed in 10% zinc formalin for 24 hours, processed, and embedded in paraffin. Sections were prepared from tissue, stained with Masson’s trichrome, and scanned using an AxioScan Z1 slide scanner (Zeiss). The extent of skin fibrosis was assessed as described (15) by measuring the distance between the epidermal-dermal junction using ZEN (Zeiss) software. An average of 20 measurements per section was obtained.

### Hydroxyproline assay and gene expression analysis.

Mouse punch skin biopsies from the injection area were flash-frozen in liquid nitrogen. The hydroxyproline assay was performed according to the manufacturer’s protocol (MilliporeSigma). To perform quantitative real-time PCR (qPCR), RNA was extracted using TRIzol or mini and microRNeasy kits (Qiagen), cDNA was synthesized using the SuperScript III First-Strand Synthesis System (Thermo Fisher Scientific), and a LightCycler 480 (Roche) with individual primer assays and SYBR Green qPCR Master Mix (Qiagen) were used. The efficiency of the primer assays is guaranteed by the manufacturer to be greater than 90%. Each reaction was performed in triplicate and analyzed using the ΔΔCt method by normalizing data against GAPDH levels. The absence of genomic DNA contamination was confirmed using control reactions lacking the reverse transcriptase enzyme during the cDNA synthesis step.

### Statistics.

Sample sizes were selected on the basis of our experience with the assays being performed to achieve sufficient power to detect biologically relevant differences in the experiments being conducted. A 1-way or 2-way ANOVA, paired or unpaired 2-tailed *t* test, and Mann-Whitney *U* test were performed where appropriate as reported in the figure legends. Parametric tests were only used on normally distributed variables as assessed by the Shapiro-Wilk test. All statistical analyses were performed using GraphPad Prism software. A comparison was considered significant if *P* was less than 0.05.

### Study approval.

Mouse studies were approved by the IACUC of UCSD in La Jolla, California, USA (protocol Bottini-S16098). Human studies were approved by the IRBs of UCSD in La Jolla, California, USA (IRB 140485) and UCSF in San Francisco, California, USA (IRB 15-16463). All individuals signed an IRB-approved consent form (UCSD IRB 140485, UCSF IRB 15-16463).

## Author contributions

NB and WP conceived the study. RZ, GSK, UH, MZ, CS, ZJH, MCL, DB, YW, ZM, SY, and ES acquired and analyzed data. FB provided essential research material. SY, ES, RP, WP, and NB supervised the research. RZ, GSK, UH, MZ, DB, ES, RP, FB, WP, and NB prepared the manuscript.

## Supplementary Material

Supplemental data

## Version 1 04/22/2022

Electronic publication
