# Supplementary material for: Oxidative stress promotes fibrosis in systemic sclerosis through stabilization of a kinase-phosphatase complex
Source: JCI Insight. 2022 Apr 22;7(8):e155761. doi: 10.1172/jci.insight.155761 (PMC9089796; doi:10.1172/jci.insight.155761)
Supplement: Supplemental data [file jciinsight-7-155761-s009.pdf]

SUPPLEMENTARY FIGURE LEGENDS

Suppl. Figure 1. PLA assay detects a complex between PTP4A1 and SRC in NHDF. (a) Representative Western blotting of lysates of NHDF stimulated with TGFβ (left) with quantification (right) of PTP4A1 expression normalized to GAPDH. N=3 lines. (b) Representative PTP4A1-SRC PLA signal in TGFβ-stimulated or unstimulated NHDF (left) with quantification (right). N=3 lines each, Two-way ANOVA with Tukey post-hoc. Dots of the same color are from the same NHDF line. Graph shows mean±SEM of data normalized to the unstimulated averages. Blue = DAPI, Magenta = PLA. Images were captured with 60x magnification. Two-tailed Welch's t test after Shapiro-Wilk. *p<0.05, **p<0.01, ****p<0.0001.

Suppl. Figure 2. PLA assay is specific for PTP4A1-SRC complex in NHDF. (a) Representative HA-SRC PLA signal in NHDF transfected with empty vector or plasmids expressing HA-tagged PTP4A1 or PTP4A2 (left) with quantification (right). N=3 lines. (b) Representative Western blotting showing PTP4A1/2 expression in NHDF from panel S2a. (c) Agarose gel run of RT-PCR using total RNA as template to quantify knockdown of PTP4A1 or 2 via ASO. (d) Representative PTP4A1-SRC PLA signal in TGFβ-stimulated NHDF incubated with PTP4A1 ASO or PTP4A2 ASO (left) with quantification (right). N=3 lines. (a, d) Blue = DAPI, Magenta = PLA. Images were captured with 60x magnification. (a) Data are shown normalized to the average values in the HA-PTP4A1 expressing NHDF. (d) Data in are shown normalized to the average values in the control ASO treatment (data not shown). (a, d) Dots of the same color are from the same NHDF line. Data shown are Mean±SEM. Two-tailed Mann-Whitney test. ****p<0.0001.

Suppl. Figure 3. PLA controls are unaffected by incubation of SScDF with TGFβ

and/or NAC. (a) Graph showing quantified PLA signal from SScDF stained with PTP4A1+Src antibodies, or with PTP4A1 antibody alone. Data is from SScDF left unstimulated or TGFβ-stimulated in the presence and absence of 20 mM NAC. N=7 lines. (b) Graph showing quantified Intracellular ROS levels detected through DCFDA staining in SScDF left unstimulated or following TGFβ stimulation with or without NAC. N=4 lines. Data shown are Mean±SEM.One-tailed unpaired t test after Shapiro-Wilk *$p<0.05$.

Suppl. Figure 4. Oxidation of PTP4A1 in HEK293T exposed to oxidative stress. Anti-PTP4A1/2 Western blotting of lysates of HEK293T cells 24 h after transfection with HA-PTP4A1 expression plasmid incubated with variable concentrations of H_2O_2 for 30 min at 37 °C. The panels show non-reducing or reducing PAGE and are representative of at least 3 experimental replicates.

Suppl. Figure 5. NMR assignment of oxidized and reduced PTP4A1. (a-c) 2D [^{1}H,^{15}N] TROSY spectrum of (a) mixed, (b) oxidized, or (c) reduced PTP4A1. (d-e) Diagrams of secondary structure propensity and chemical shift index based on the NMR assignment of (d) oxidized PTP4A1, in which 94.1% of residues were assigned or (e) reduced PTP4A1, in which 86.1% of residues were assigned.

Suppl. Figure 6. Covalently immobilized PTP4A1 co-precipitates SRC, SRC SH3SH2 and SH2 alone. (a) Representative Western blotting of binding of purified full-length SRC to PTP4A1 covalently attached to NHS-activated Agarose (left) with quantification (right) (n=5). NHS-activated Agarose-bound BSA was used as a negative control. (b) Representative Western blotting of binding of GST-tagged SRC SH2 to Ni-NTA Agarose-bound His_6-tagged PTP4A1 (left) with quantification (right) (n=5). GST alone was used as a negative control. (c-d) Representative Western blotting (left) with quantification (right)

(n=5) of (c) binding of GST-tagged SRC SH2, or (d) GST-tagged SRC SH3SH2, to covalently immobilized PTP4A1. The ratio of GST-tagged protein to GST alone in each independent experiment is shown in the graphs in (c) and (d). (a-d) Two-tailed paired t test. **p<0.01, ***p<0.001.

Suppl. Figure 7. NMR assessment of PTP4A1 bound to SRC SH3SH2. 2D [^{1}H,^{15}N] TROSY spectrum of (a) oxidized, or (b) reduced PTP4A1 in the presence of increasing amounts (color-coded as indicated) of SRC SH3SH2.

Suppl. Figure 8. NMR assignment of SRC SH3SH2 and assessment of SRC SH3SH2 bound to PTP4A1. (a) Diagrams of secondary structure propensity and chemical shift index based on the NMR assignment of SRC SH3SH2. (b-c) 2D [^{1}H,^{15}N] TROSY spectrum of SRC SH3SH2 in the presence of increasing amounts (color-coded as indicated) of (b) oxidized or (c) reduced PTP4A1.

Suppl. Figure 9. NMR-based docking model of oxidized PTP4A1 binding to SRC SH3SH2. The lowest energy HADDOCK model of oxidized PTP4A1-SRC SH3SH2 in ribbon and solvent accessible surface representation. The contact residues of oxidized PTP4A1 (blue) and SRC SH3SH2 (green) were highlighted respectively.

Suppl. Figure 10. Assessment of SRC mutant phosphorylation by phos-tag gel and immunoblotting. (a-b) Representative Western blotting showing that (a) autophosphorylation or (b) CSK phosphorylation of WT SRC is unspecific for Y^{416} or Y^{527}. (c-f) Phos-tag gel patterns and representative Western blotting images of (c-d) autophosphorylation time course of SRC^{Y527F} or (e-f) phosphorylation of SRC^{K295A} by CSK. The lower MW unshifted band in panel e (arrow) is CSK.

Suppl. Figure 11. TBRI^{CA} treatment induces and fibroblast-specific *Ptp4a1* KO reduces skin fibrosis in C567BL7/6 mice. Mice were injected with PBS (control) or with 6.67 × 10^7 pfu adenovirus. (a) Representative images of skin specimens from TBRI^{CA}-treated or control C57BL/6 mice stained with Masson's Trichrome (n = 3 mice/group). Yellow lines show representative quantification of dermis layer thickness. At least 20 measurements were taken across each section. (b-c) Quantification of (b) the thickness of dermis layer and (c) collagen levels using hydroxyproline assay in skin specimens from panel S11a. An average of 20 measurements were taken across each section. (d-g) Inducible *Ptp4a1* KO was driven by the COL1A1 promoter in the TBRI^{CA} model before inducing fibrosis (at least 10 mice/group). (d) Representative Masson's Trichrome staining. (e-f) Quantification of (e) dermal thickness and (f) collagen levels using hydroxyproline assay in skin specimens from panel S11d. (e) Dotted line shows average normal dermal thickness (g) Relative expression levels of *Ptp4a1*, *Acta2*, *Col1a1* and *Tgfβr1* were shown. All data are presented as mean±SEM. (a, d) Images were captured with 20x magnification. (b, c, and f) Two-tailed Welch's t test after Shapiro-Wilk, two-tailed Mann-Whitney (e, g), *p<0.05, **p<0.01, ***p<0.001.

Suppl. Figure 12. Global *Ptp4a1* KO prevents and mitigates progression of fibrosis in TBRI^{CA} model. (a) Representative PLA signal in skin specimens from mice shown in UBC-Cre mice treated with tamoxifen before induction of fibrosis (left) with quantification (right) (n=5). (b-c) mRNA measured via qPCR on specimens in which knockout was induced before fibrosis (b) or in established fibrosis (c). (a) Blue = DAPI, Magenta = PLA. Images were captured with 20x magnification. (a-c) All data are presented as Mean±SEM. Two-tailed Mann-Whitney. *p<0.05, **p<0.01, ***p<0.001.

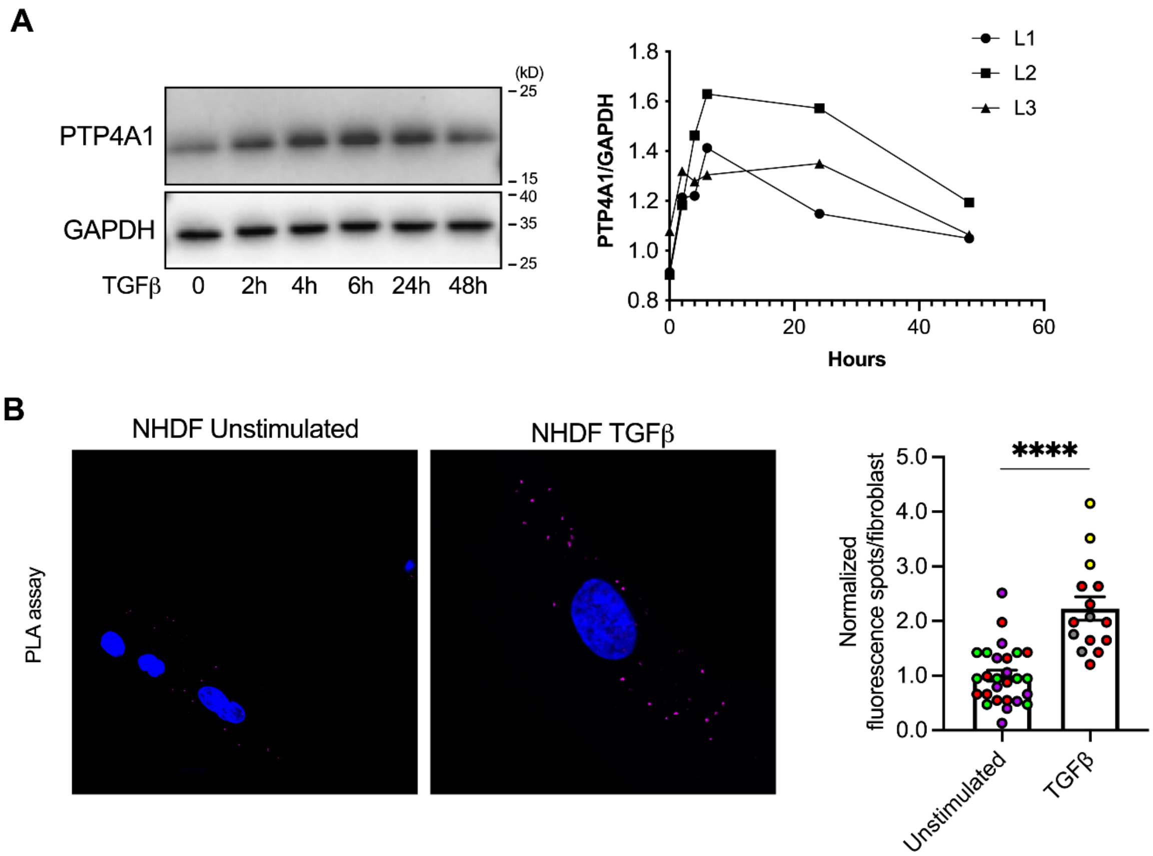

Supplementary Figure 1

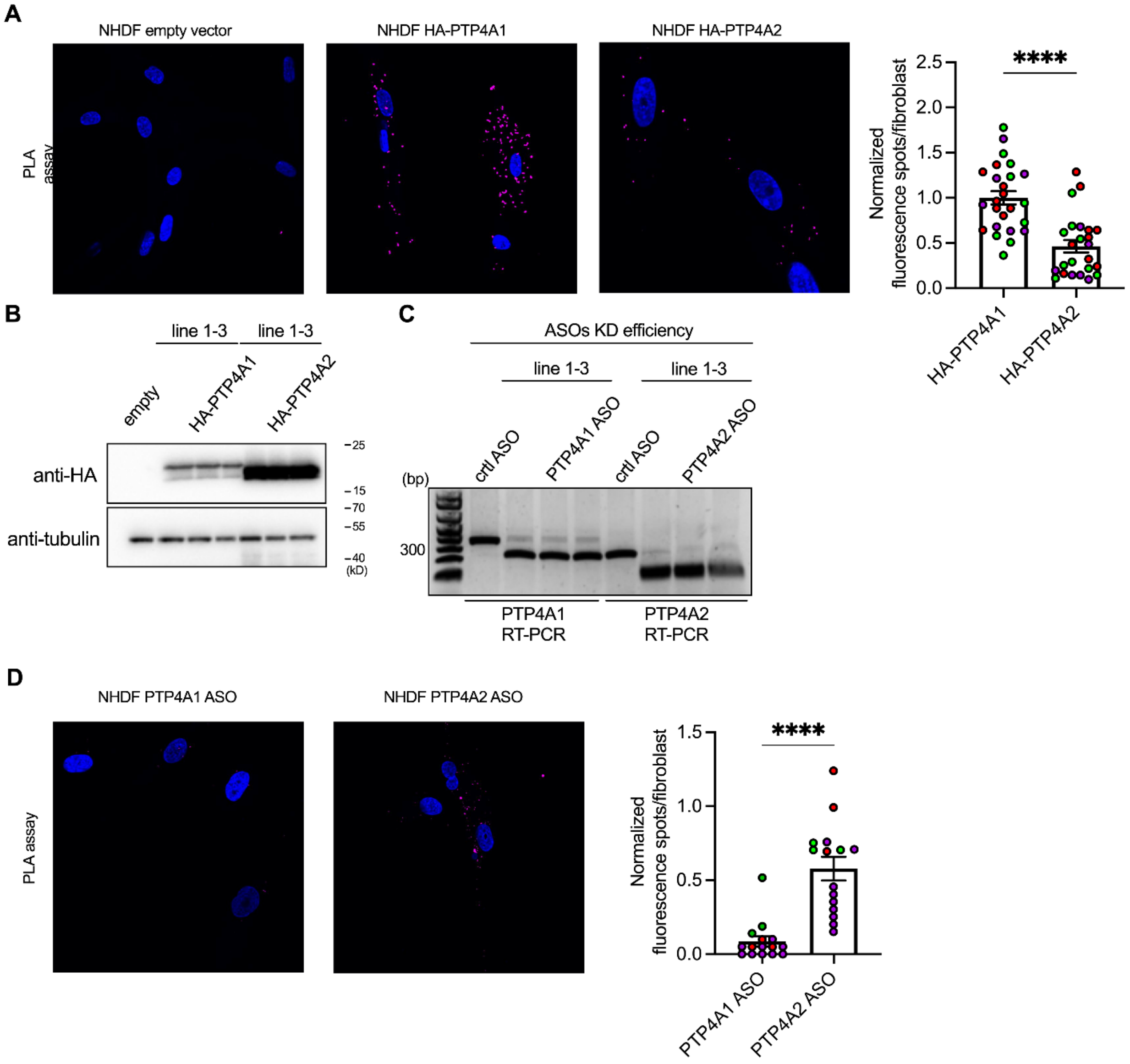

Supplementary Figure 2

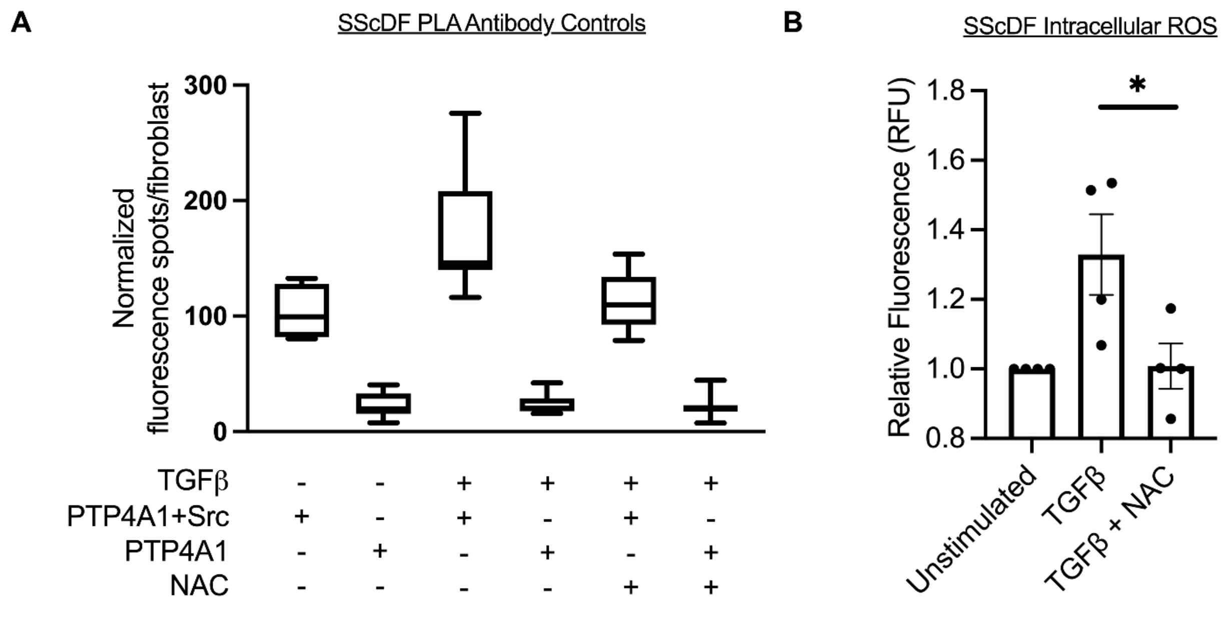

Supplementary Figure 3

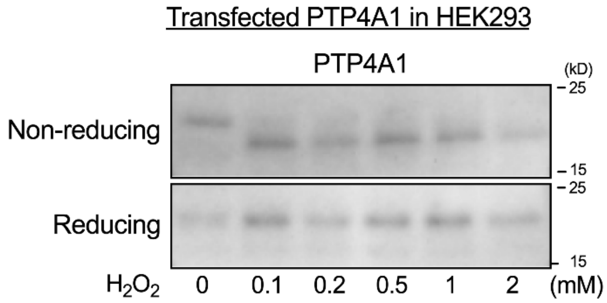

Supplementary Figure 4

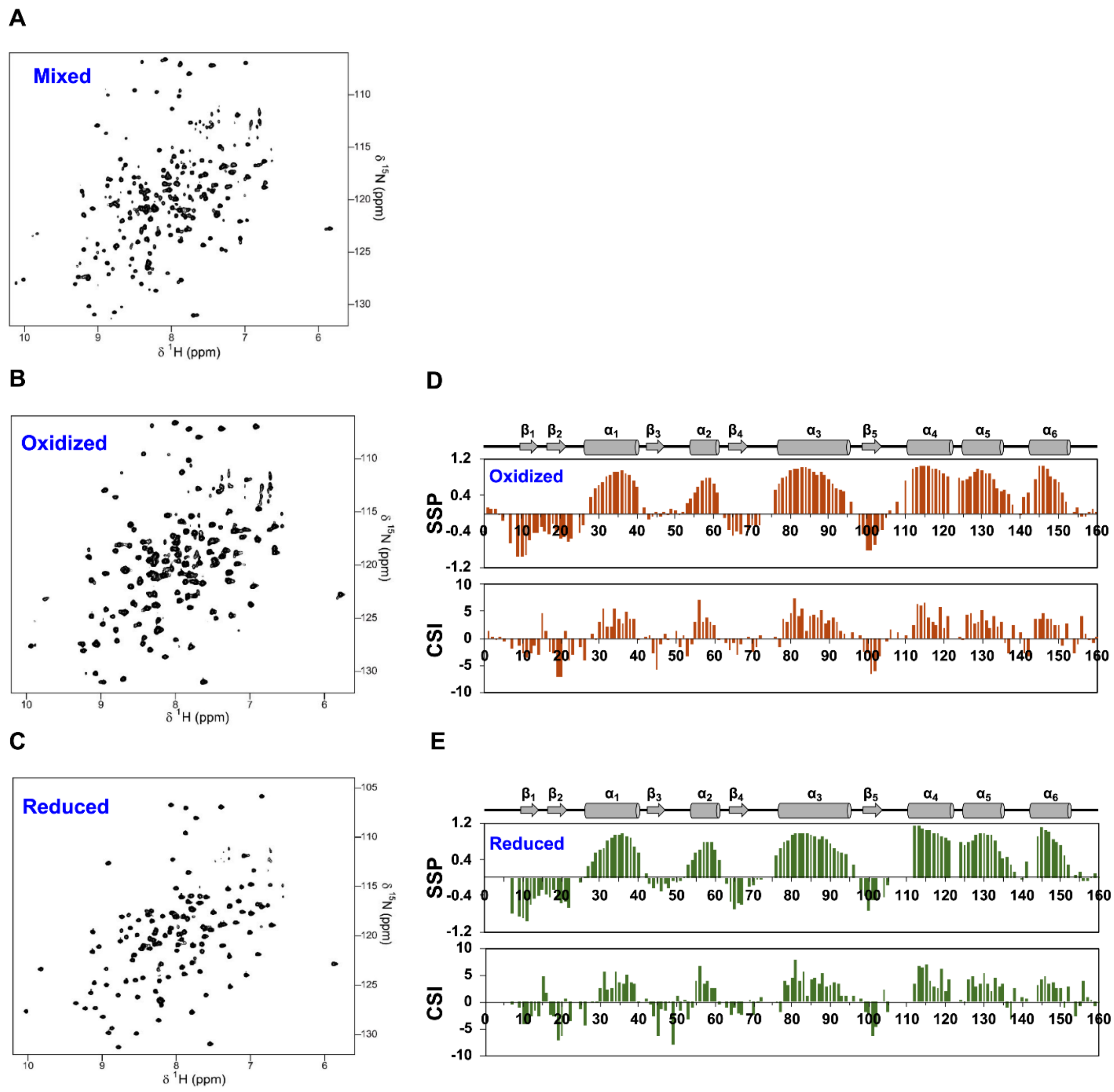

Supplementary Figure 5

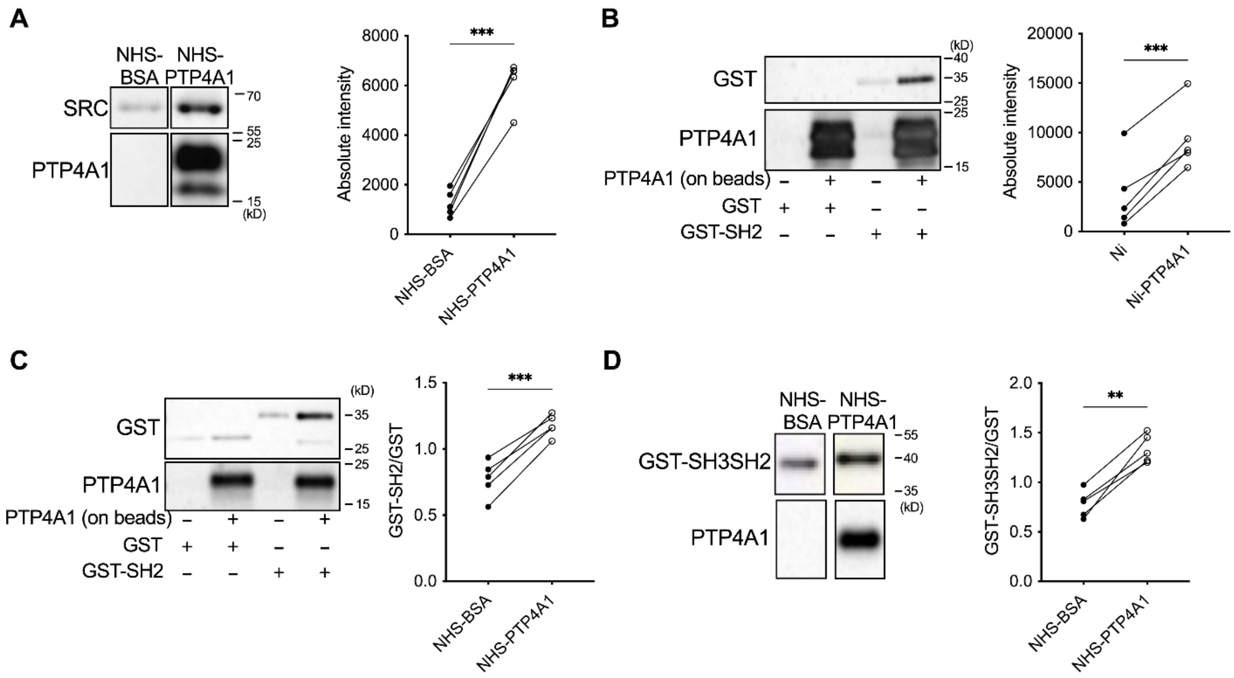

Supplementary Figure 6

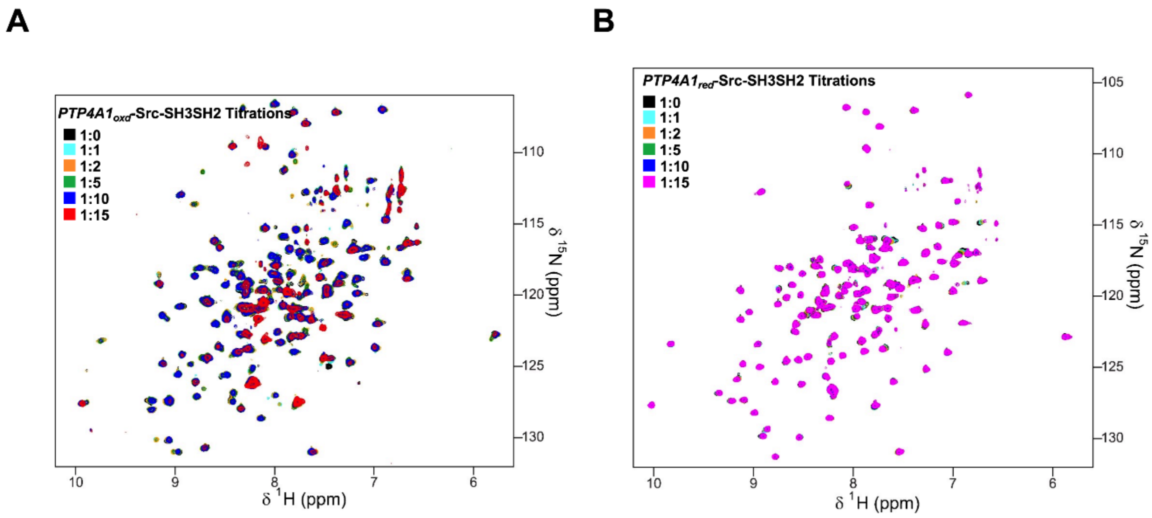

Supplementary Figure 7

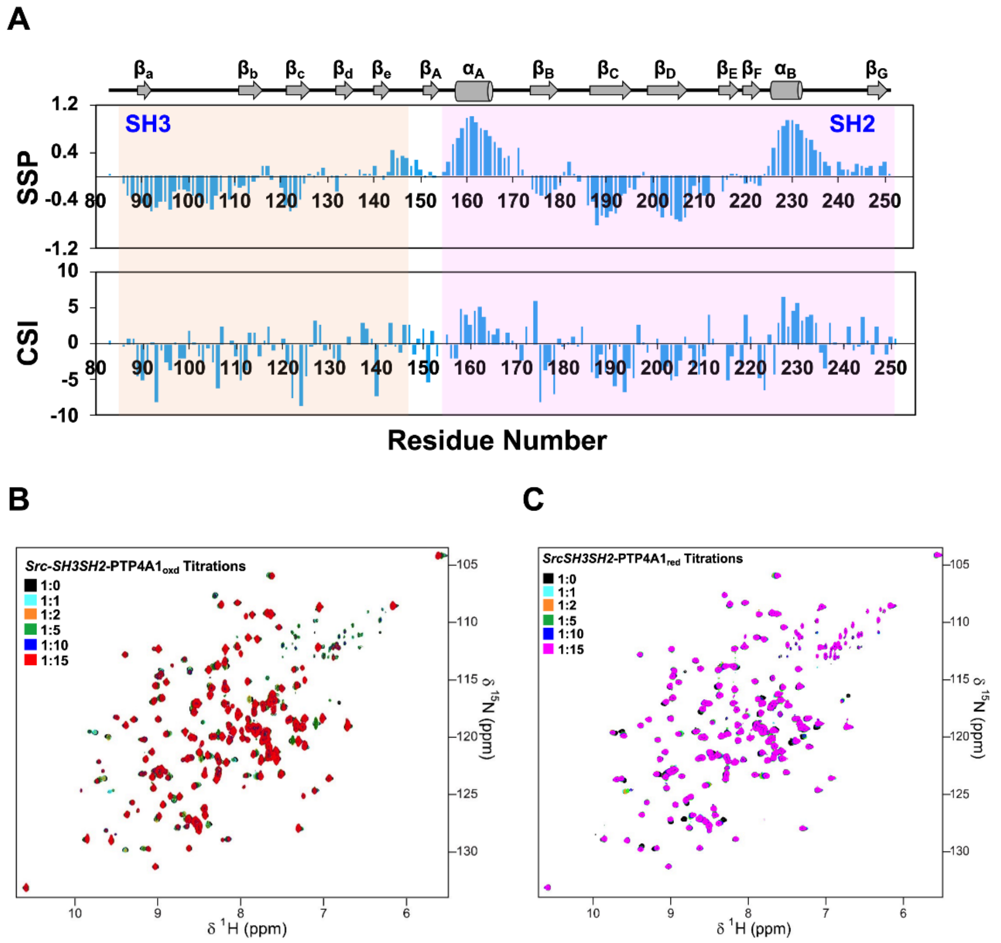

Supplementary Figure 8

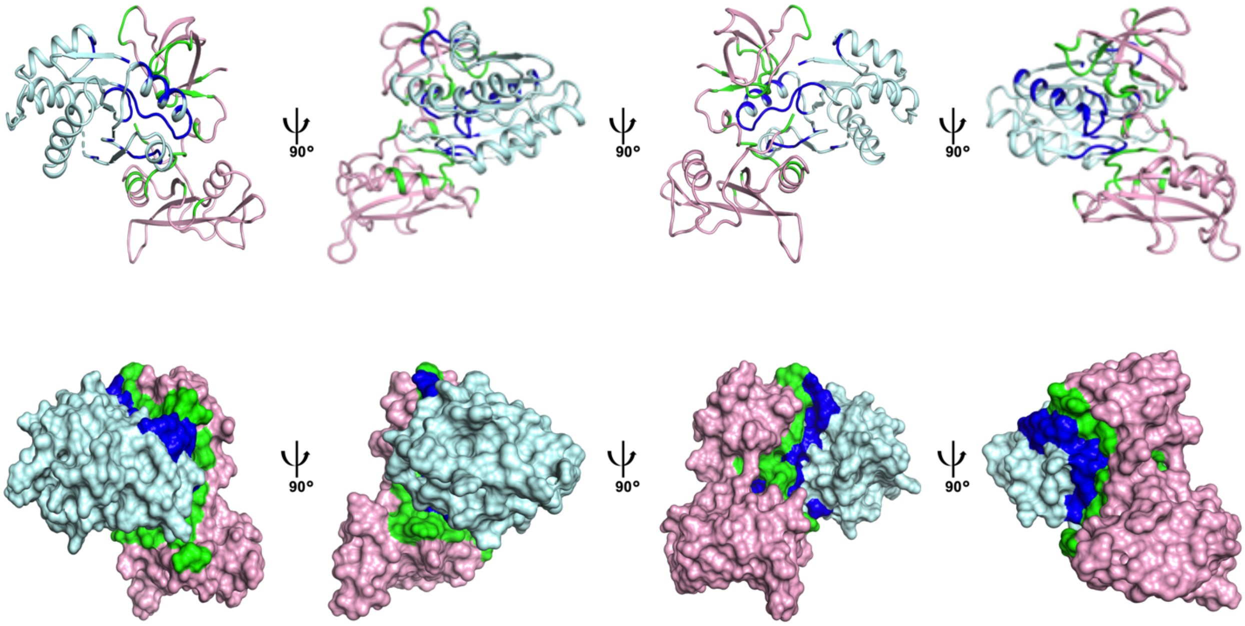

Supplementary Figure 9

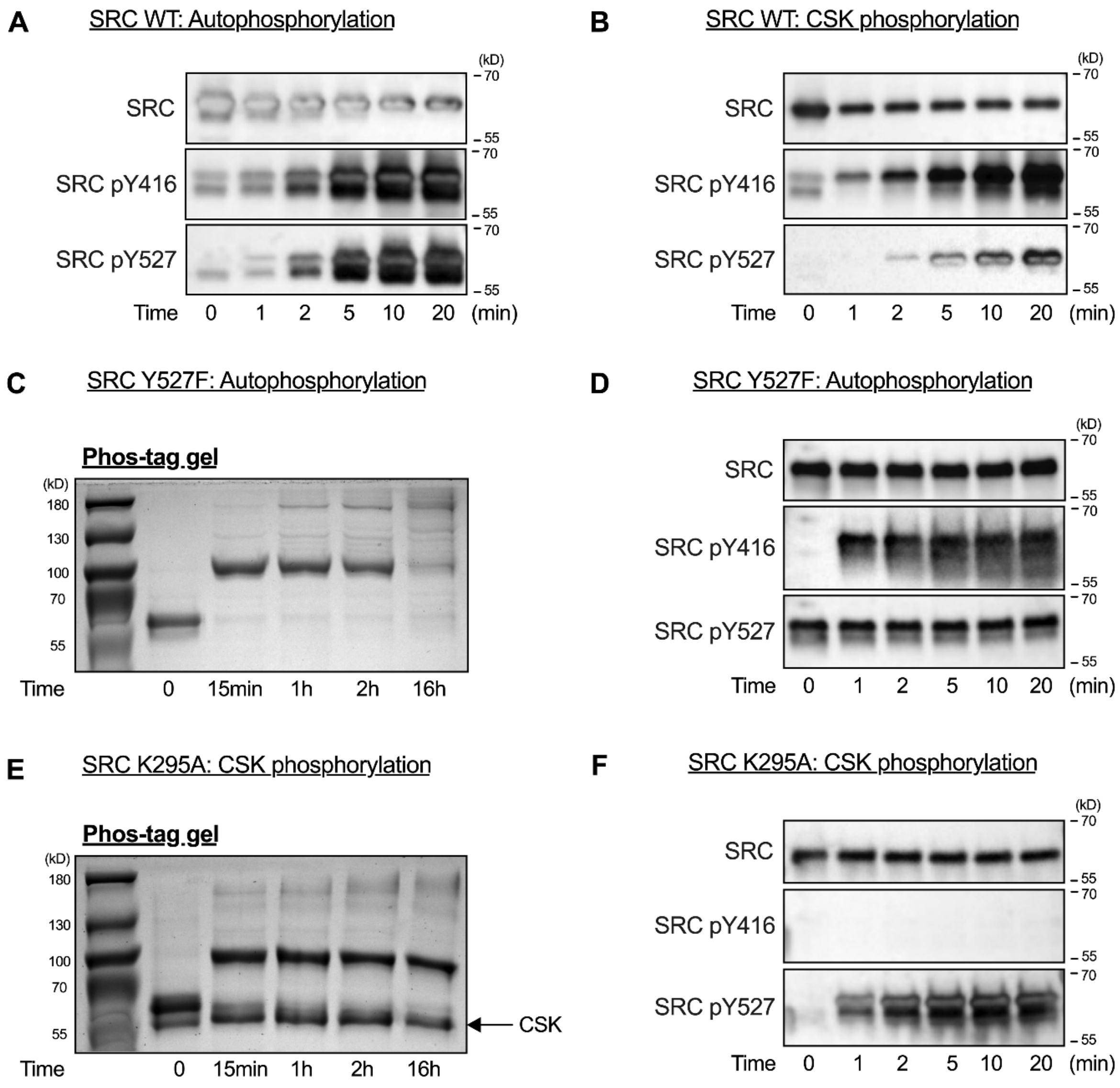

Supplementary Figure 10

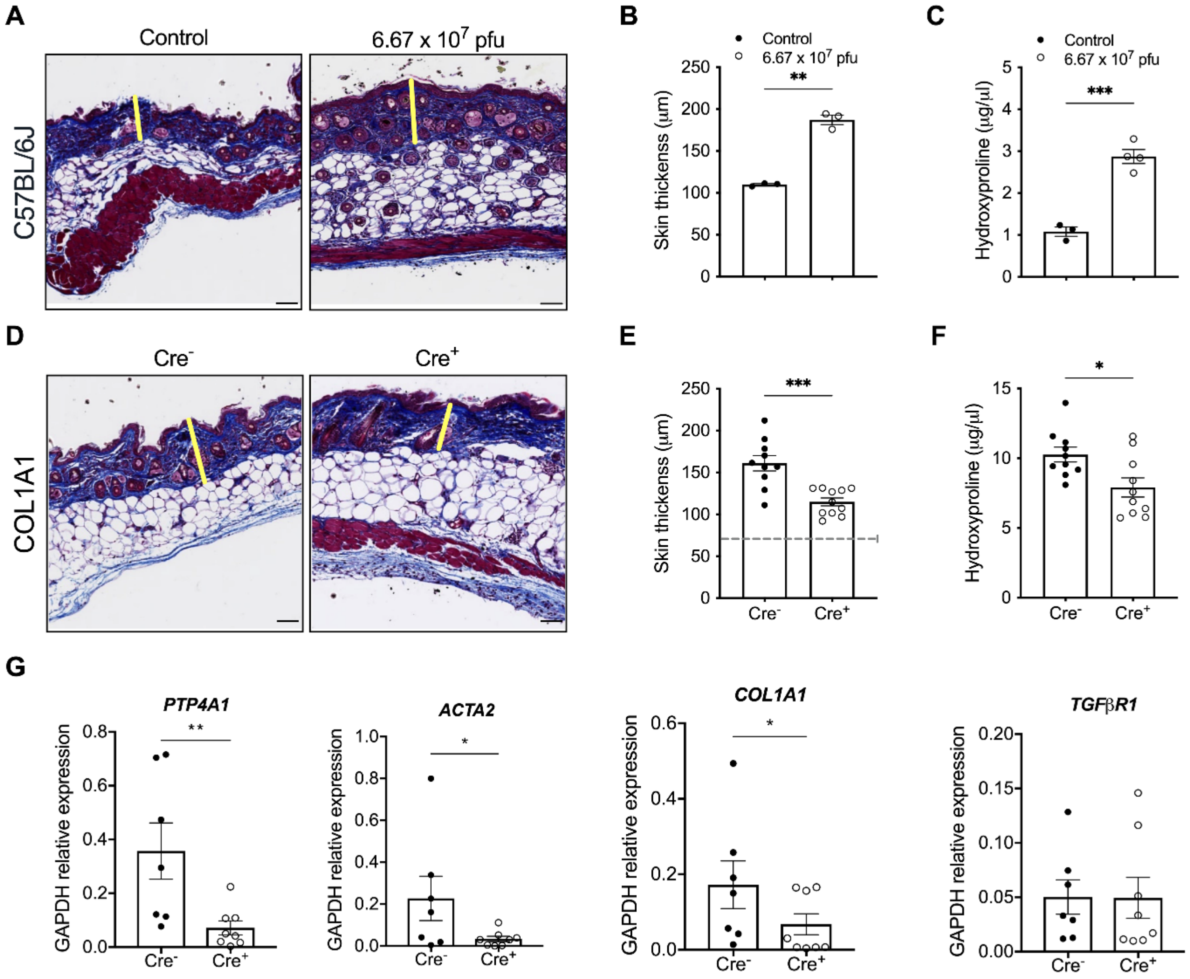

Supplementary Figure 11

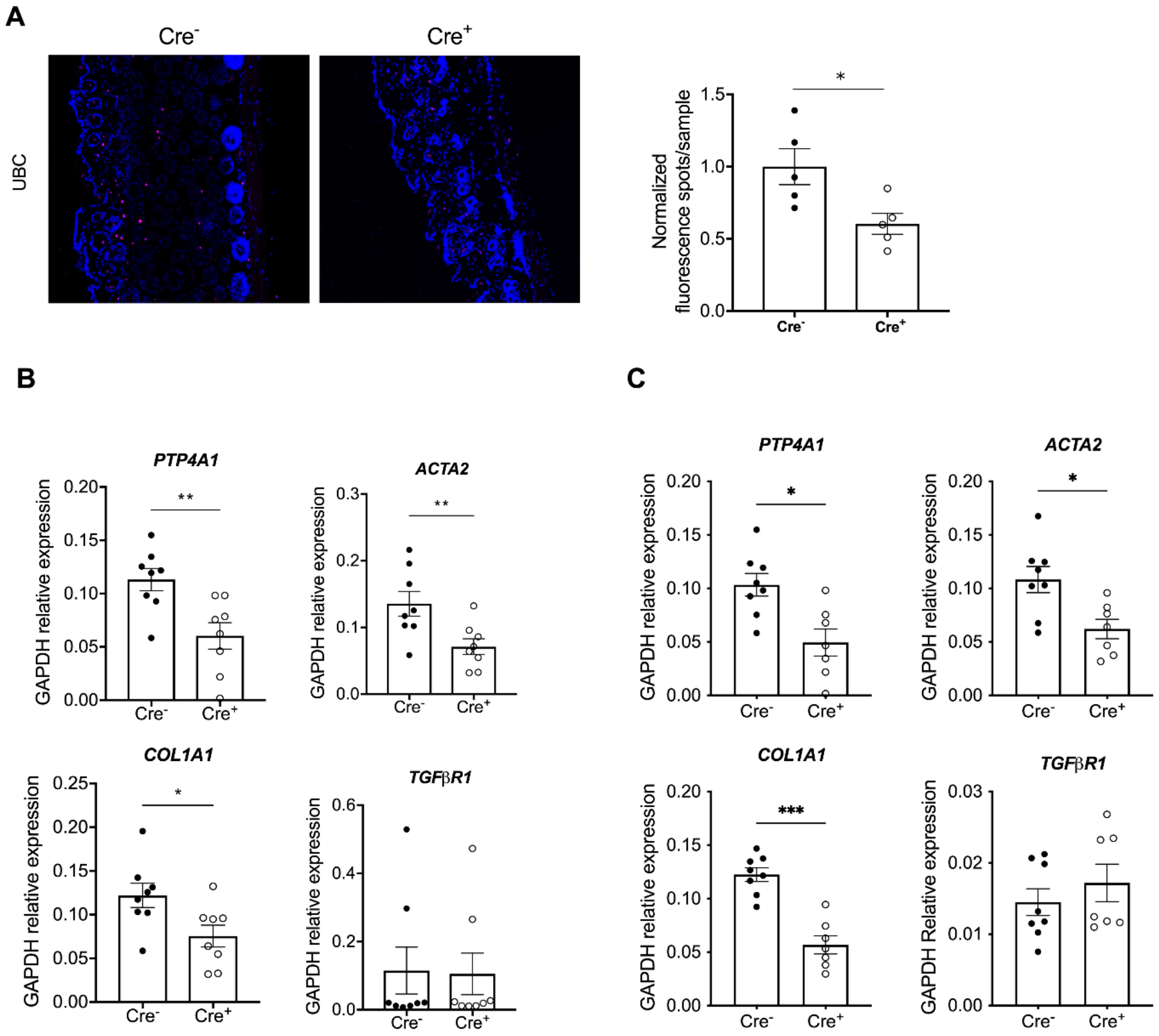

Supplementary Figure 12

Suppl. Table 1. Clinical features of SSc patients.

PLA assay performed on SScDF

Patient ID	Age at onset	Sex	Race	Phenotype	*mRSS score	Immunosuppressants
1	37	Female	White	Diffuse	20	No
2	45	Female	White	Diffuse	15	No
3	58	Female	White	Diffuse	40	No
4	21	Female	White	Diffuse	26	No
7	52	Female	White	Diffuse	14	Yes
8	23	Female	White	Diffuse	20	Yes
9	41	Female	Asian	Diffuse	3	Yes
10	35	Male	White	Diffuse	18	No

PLA assay performed on skin biopsies

Patient ID	Age at onset	Sex	Race	Phenotype	mRSS score	Immunosuppressants
3	69	Male	White	Diffuse	26	No
6	57	Female	White	Diffuse	6	Yes
7	40	Male	African American	Diffuse	10	No
8	58	Female	White	Diffuse	14	Yes
10	62	Male	African American	Diffuse	31	No
14	53	Female	White	Diffuse	20	No
17	51	Male	White	Diffuse	23	No

*mRSS score: Modified Rodnan Skin Score (1 to 51)

PLA assay performed on NHDF

NHDF #	Age at Isolation	Sex	Race	Source
1	34	Female	White	ATCC
2	41	Female	White	ATCC
3	40	Female	White	NDRI
4	25	Female	White	NDRI
9	36	Female	White	UCSD
10	26	Male	White	UCSD
